# Chronic oral administration of ibrutinib prevents long-term memory deficits and reduces AD pathology and neuroinflammatory responses in a mouse model of AD

**DOI:** 10.1186/s13041-025-01225-7

**Published:** 2025-07-21

**Authors:** Hyun-ju Lee, Sora Kang, Yoo Joo Jeong, Jin-Hee Park, Jeong-Woo Hwang, Chan-Hu Gu, Tae-Mi Jung, Seokjun Oh, Ji-Yeong Jang, Hyang-Sook Hoe

**Affiliations:** 1 Department of Neural Development and Disease, Korea Brain Research Institute (KBRI), 61, Cheomdan-ro, Daegu, 41062 Republic of Korea; 2 AI-based neurodevelopmental diseases digital therapeutics group, Korea Brain Research Institute (KBRI), 61, Cheomdan-ro, Daegu, 41062 Republic of Korea; 3 Department of Brain and Cognitive Sciences, Daegu Gyeongbuk Institute of Science & Technology, Daegu, 42988 Republic of Korea

**Keywords:** Ibrutinib, Aβ, Tau, Cognitive function, Neuroinflammation

## Abstract

We previously demonstrated that ibrutinib has therapeutic efficacy against AD pathologies when injected intraperitoneally at a lower dosage (10 mg/kg, daily for 2 weeks) or orally at a higher dosage (30 mg/kg, daily for 1 month) in AD mice models. However, the effect of chronic lower dose of ibrutinib by oral administration on AD pathologies has not been investigated yet. Therefore, we investigated whether long-term oral administration of ibrutinib at a lower dose (1 or 10 mg/kg, daily for 5 months) on AD pathology and in vivo toxicity in 5xFAD mice. We found ibrutinib enhanced cognitive function and alleviated Aβ pathology in 5xFAD mice without hepatotoxicity. Furthermore, ibrutinib-treated 5xFAD mice decrease tau hyperphosphorylation, p-GSK3α/β levels, and markers of neuroinflammation such as Iba-1, GFAP, and NLRP3. Collectively, these translational studies indicate chronic oral administration of ibrutinib at low doses improves cognitive function and suppresses AD pathology/neuroinflammation in an AD mice model thereby having potential as an effective multitarget AD therapeutic in clinical application.

**Supplementary Information:**

The online version contains supplementary material available at 10.1186/s13041-025-01225-7.

Alzheimer’s disease (AD) has the highest prevalence and incidence among neurodegenerative diseases [1]. Two prominent hallmarks of AD are the abnormal accumulation of extracellular amyloid beta (Aβ) plaques and intracellular neurofibrillary tangles (NFTs). The formation of these protein aggregates is accompanied by exacerbation of neuroinflammation which leads to neuronal deterioration and subsequent cognitive deficits [2]. Given the heterogeneity of major diagnostic symptoms, Aβ/tau pathology, memory impairments, and neurological dysfunction among AD patients, single-target drugs are unlikely to be sufficient therapies. An alternative route to efficient therapeutic strategies for AD may be to combine two or more drugs targeting distinct pathological pathways or to develop multitarget drugs.

The search for AD therapeutics is also increasingly turning toward drug repurposing, in which approved drugs are used for novel indications. Cancer and AD share several risk factors and pathological mechanisms, suggesting potential applications of anticancer drugs in AD treatment [3]. We previously demonstrated that protein kinase inhibitors ameliorate multiple AD pathologies, including Aβ/tau pathology, memory impairments, neuroinflammation and/or synaptic dysfunction [4, 5]. Specifically, ibrutinib, a Bruton’s tyrosine kinase (BTK) inhibitor approved by the FDA for chronic lymphocytic leukemia, exhibits therapeutic efficacy against deficits in cognitive/synaptic function and AD pathology in AD mice when injected intraperitoneally at a lower dosage (10 mg/kg, i.p., daily for 2 weeks) or by oral gavage at a higher dosage (30 mg/kg, p.o., daily for 1 month) [6]. Moreover, the therapeutic effects of against AD pathologies in 5xFAD mice are superior to those of other tyrosine kinase inhibitors, including cabozantinib (VEGFR2 inhibitor) and PD180970 (Bcr-Abl inhibitor) [7]. For repurposing anticancer drugs as an AD treatment, anticancer drugs should be administered at lower doses compared to cancer-treating dose to minimize cytotoxicity. For example, nilotinib, an anticancer drug for Ph + chromosome chronic myeloid leukemia significantly improved cognitive function or reduced amyloid burdens when treated at lower doses compared to cancer-treating dose for over 6 months in AD patients [8]. However, we did not investigate whether chronic oral administration of a lower dose (1 or 10 mg/kg, p.o., daily for 5 months) of ibrutinib modulates AD pathologies. Data from such studies are necessary for dose optimization in preparation for phase II clinical trials of anticancer drugs repurposed as AD treatments as currently prescribed AD drugs (e.g., donepezil and memantine) must be administered over 4 months for therapeutic efficacy [9, 10].

To address this gap, we investigated the effects of long-term oral injection of ibrutinib at a lower dose in AD pathologies of 5xFAD mice. Here, we further demonstrated that chronic oral administration of ibrutinib at lower dose (10 mg/kg, p.o., daily for 5 months) enhanced spatial memory and recognition memory in 5xFAD mice (Fig. 1A-C). In addition, we investigated whether ibrutinib itself can improve cognitive function in WT mice and found that oral administration of 1 or 10 mg/kg ibrutinib (daily for 2 weeks) did not alter short-term and recognition memory in WT mice compared with vehicle-treated WT mice (Supplementary Fig. 1). BTK and epidermal growth factor receptor (EGFR), which are an on-target and major off-target of ibrutinib, respectively, are closely associated with memory decline in age-related pathology and AD. For example, upregulation of BTK expression is observed in the brains of AD patient’s postmortem, and inhibition of BTK improves spatial memory in progeroid mice model [11, 12]. Similarly, astrocytic expression of EGFR is increased in the brains of AD patients, and inhibition of EGFR restores cognitive deficits in APP/PS1 mice [13]. Therefore, the memory-restoring effects of chronic oral administration of ibrutinib at lower doses in AD mouse models may reflect on-target and/or off-target therapeutic effects. In future work, we will investigate whether chronic oral treatment of ibrutinib improves cognitive function in 5xFAD mice in a BTK- or EGFR-dependent manner.

**Fig. 1:**
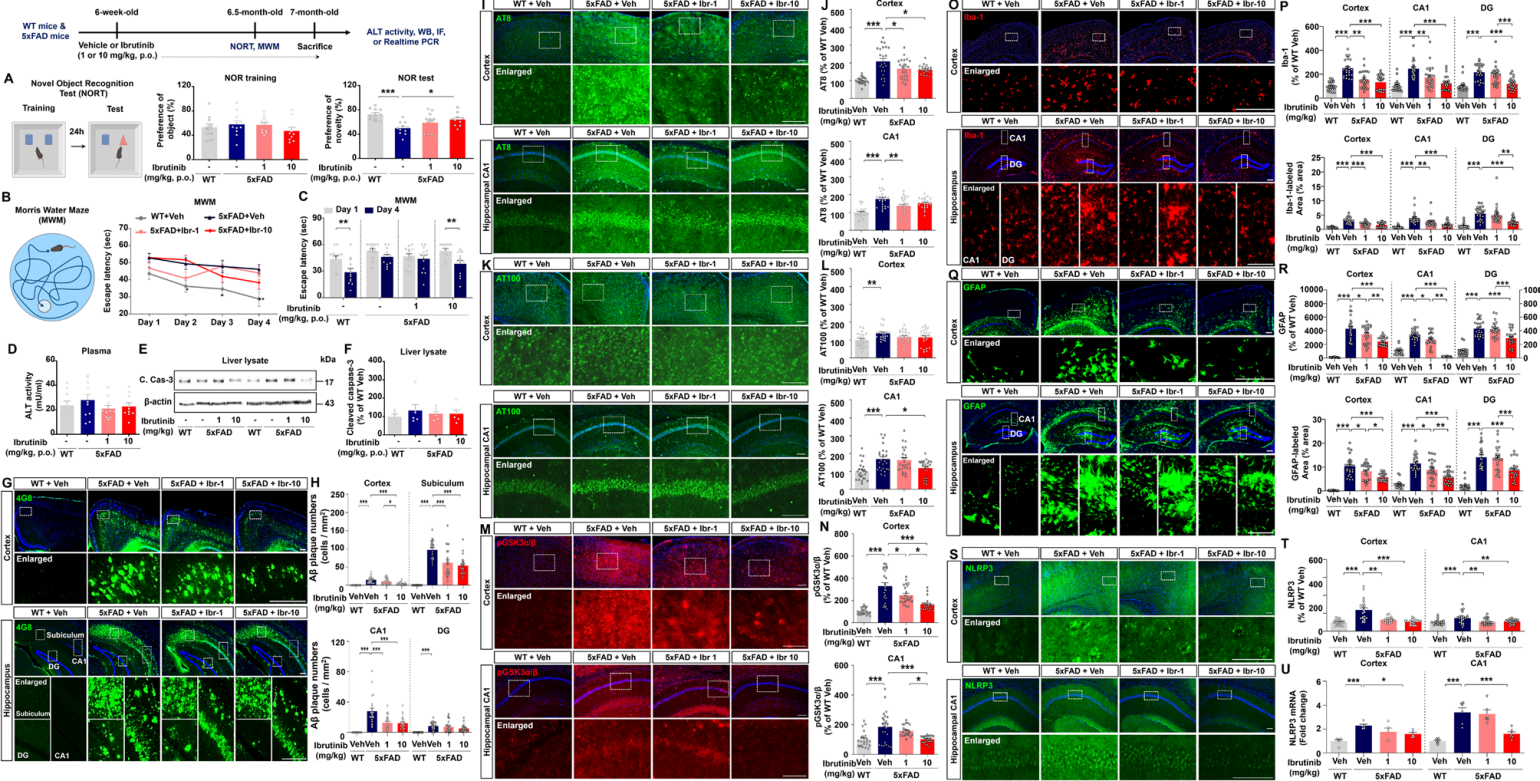
Chronic oral administration of ibrutinib enhances long-term memory and reduces Aβ pathology, tau phosphorylation, and gliosis in 5xFAD mice. WT mice and 5xFAD mice (6 weeks old, male) were administered vehicle (5% DMSO + 30% PEG + 5% Tween 80 + 60% ddH_2_O) or ibrutinib (1 or 10 mg/kg, p.o.) by oral gavage daily for 5 months. (**A**-**C**) Recognition and spatial memory were evaluated by novel object recognition (NOR) test and Morris water maze (MWM) tests, respectively (*n* = 10–13 mice/group). (**D**-**F**) The hepatotoxicity of ibrutinib (1 or 10 mg/kg, p.o., daily for 5 months) was evaluated by measuring plasma ALT activity (**D**, *n* = 10 mice/group) and by western blot analysis of cleaved caspase-3 in liver lysates (**E**-**F**, *n* = 6 mice/group). (**G**-**H**) IF staining of mouse brain slices with an anti-β-Amyloid_17–24_ (4G8) antibody (*n* = 24 brain slices from 6 mice/group). Scale bar = 100 μm for the cortex and 200 μm for the hippocampus. (**I**-**N**) IF staining of mouse brain slices with anti-pTau^Ser202/Thr205^ (AT8) (**I**), or anti-pTau^Thr212/Ser214^ (AT100) (**K**), or an anti-p-GSK3 α/β (**M**) antibodies (*n* = 21–24 brain slices from 6 mice/group). Scale bar = 100 μm. (**O**-**T**) IF staining of mouse brain slices with anti-Iba-1 (**O-P**), anti-GFAP (**Q-R**), or anti-NLRP3 (**S-T**) (*n* = 23–24 brain slices from 6 mice/group). (**U**) Real-time PCR of NLRP3 mRNA expression in the cortex and hippocampus of WT and 5xFAD mice treated as described above (*n* = 5–6 mice/group). (**O**, **Q**) Scale bar = 200 μm. (**S**) Scale bar = 100 μm. *p < 0.05, **p < 0.01, and ***p < 0.001

After the behavioral tests, we assessed plasma alanine aminotransferase (ALT) activity and liver cleaved caspase-3 expression to determine whether chronic oral administration of ibrutinib causes in vivo hepatotoxicity. Compared with vehicle treatment, chronic oral administration of ibrutinib at lower doses did not alter plasma ALT activity or liver cleaved caspase-3 expression in 5xFAD mice (Fig. 1D-F). Additionally, we evaluated whether daily oral administration of ibrutinib for 2 weeks induces liver toxicity in WT mice. We found that oral dosing with 1 or 10 mg/kg ibrutinib did not affect plasma ALT activity or liver cleaved caspase-3 expression in WT mice compared with vehicle-treated WT mice (Supplementary Fig. 2). These data indicate that treatment of ibrutinib rescued cognitive function without causing hepatotoxicity in a mouse model of AD and/or WT mice. However, a limitation of this study is the lack of data on potential hepatotoxicity following chronic administration of ibrutinib (e.g. consecutive 5 months) in WT mice. Thus, future studies will address the long-term safety profile of ibrutinib, including liver toxicity, which will have important implications for its clinical repurposing.

Senile plaques, a major hallmark of AD, are produced by amyloidogenic proteolytic processing of amyloid precursor protein (APP), and their accumulation leads to neuronal excitotoxicity, oxidative stress, synaptic dysfunction and, ultimately, memory decline [14–16]. Here, we found that chronic oral administration of ibrutinib significantly reduced Aβ plaque deposition in 5xFAD mice (Fig. 1G-H). These data indicate that chronic oral administration of ibrutinib at 10 mg/kg more effectively alleviates amyloidopathy in 5xFAD mice.

Another major hallmark of AD is tau hyperphosphorylation by tau kinases such as GSK-3α/β, CDK5, and DYRK1A. GSK3-α/β is highly expressed in the brains of AD patients and AD mouse models [17]. Moreover, GSK3 overexpression contributes to astrocyte activation and cognitive defects, indicating a direct association between GSK3α/β and tauopathy in AD pathoprogression [18, 19]. We previously reported that short-term ibrutinib treatment (10 mg/kg, daily for 2 weeks, i.p.) reduces tau phosphorylation and suppresses tau kinase p-CDK5 levels without altering DYRK1A in a mouse model of AD [6, 7]. In the present study, we found that the upregulated tau hyperphosphorylation at Ser202/Thr205 residues (detected by AT8) and at Thr212/Ser214 residues (detected by AT100) in vehicle-treated 5xFAD mice (compared with vehicle-treated WT mice) was decreased upon chronic oral administration of ibrutinib in the 5xFAD mice (Fig. 1I-L). We then explored the effects of ibrutinib on tau kinase and found that the increase in p-GSK3α/β levels observed in vehicle-treated 5xFAD mice (compared with vehicle-treated WT mice) was significantly suppressed by chronic oral administration of ibrutinib-treated 5xFAD mice (Fig. 1M-N). Based on the previous and the present study, it is suggested that ibrutinib downregulates tau phosphorylation by inhibiting tau kinases CDK5 and GSK-3α/β phosphorylation but not DYRK1A. Interestingly, in AD pathoprogression, Aβ stimulates EGFR phosphorylation, and downstream signaling activates GSK-3β via phosphorylation at Y216 (an active site) [20]. In addition, an in vitro study suggested that amyloid β oligomers can bind EGFR [21]. Taken together, these observations suggest that ibrutinib inhibits Aβ-activated EGFR (off-target of ibrutinib), which suppresses GSK-3β phosphorylation at Y216 and subsequently attenuates tau hyperphosphorylation in a mouse model of AD. Future work will examine whether the interaction between Aβ and EGFR to alter tau hyperphosphorylation and whether ibrutinib regulates tauopathy in an EGFR-dependent manner in mouse models of AD.

In AD pathology, exacerbated Aβ/tau pathology accelerate gliosis and neuroinflammation and vice versa. Therefore, we further investigated the effect of chronic oral administration of ibrutinib on gliosis in 5xFAD mice. We found that the upregulation of microgliosis (Iba-1) and astrogliosis (GFAP) in vehicle-treated 5xFAD mice was significantly reduced by chronic oral administration of ibrutinib-treated 5xFAD mice (Fig. 1O-R, Supplementary Fig. 3). Our previous reports and the present study show that ibrutinib ameliorates Aβ- and/or tau-mediated gliosis and proinflammatory cytokine release in various AD mouse models [7, 22]. These findings raise the following question: how does ibrutinib alter neuroinflammatory responses in AD mice model? It is reported that NLRP3 inflammasome activation is the predominant molecular mechanism of the excessive inflammatory response in AD pathoprogression [23]. Conversely, inhibiting the NLRP3 inflammasome attenuates cognitive impairment and/or microgliosis in a mouse model of AD [24–26]. Interestingly, in a mouse model of brain ischemia, ibrutinib inhibits NLRP3/caspase-1/IL-1β upregulation by suppressing BTK [27]. The present study is the first to demonstrate that chronic ibrutinib treatment attenuates NLRP3 upregulation in 5xFAD mice (Fig. 1S-T). We further examined the effect of chronic oral administrations of ibrutinib on NLRP3 mRNA levels and found that vehicle-treated 5xFAD mice significantly increased NLRP3 mRNA levels in the cortex and hippocampus compared to vehicle-treated WT mice (Fig. 1U). Importantly, 10 mg/kg ibrutinib chronic administration significantly decreased NLRP3 mRNA levels in the cortex and hippocampus of 5xFAD mice (Fig. 1U). However, we have not examined whether ibrutinib-treated 5xFAD mice modulates the expression of the components of NLRP3 inflammasome activation (e.g., ASC and caspase-1). Moreover, we cannot exclude the possibility that ibrutinib modulates another neuroinflammation-associated molecular target to reduce neuroinflammation in 5xFAD mice. Thus, these possibilities will be addressed using shRNA systems in future study.

In conclusion, the present study demonstrates that chronic oral administration of ibrutinib at a lower dose (10 mg/kg, p.o., daily for 5 months) prevents cognitive impairments and attenuates senile plaque deposition, tau hyperphosphorylation, and gliosis without inducing hepatotoxicity in 5xFAD mice. Given that anticancer drugs need to be prescribed at lower doses compared to cancer-treating dose for elucidating AD therapeutic efficacy and minimizing cytotoxicity [8], the present findings suggest that ibrutinib is suitable for clinical application.

## Electronic supplementary material

Below is the link to the electronic supplementary material.

Supplementary Material 1

## Data Availability

No datasets were generated or analysed during the current study.
