# Supplementary material for: Chronic oral administration of ibrutinib prevents long-term memory deficits and reduces AD pathology and neuroinflammatory responses in a mouse model of AD
Source: Mol Brain. 2025 Jul 21;18:65. doi: 10.1186/s13041-025-01225-7 (PMC12281740; doi:10.1186/s13041-025-01225-7)
Supplement: Supplementary file 1 — Supplementary Material 1 [file 13041_2025_1225_MOESM1_ESM.docx]

**Chronic oral administration of ibrutinib prevents long-term memory deficits and reduces AD pathology and neuroinflammatory responses in a mouse model of AD**

**Hyun-ju Lee^1,2,*^, Sora Kang^1,2^, Yoo Joo Jeong^1,2,3^, Jin-Hee Park^1^, Jeong-Woo Hwang^1,2^,** **Chan-Hu Gu^2^, Tae-Mi Jung^1,2^, Seokjun Oh^1,2^, Ji-Yeong Jang^1,2,3^, Hyang-Sook Hoe^1,2,3*^**

^1^ Department of Neural Development and Disease, Korea Brain Research Institute (KBRI), 61, Cheomdan-ro, Daegu 41062, Republic of Korea; ^2^AI-based neurodevelopmental diseases digital therapeutics group, Korea Brain Research Institute (KBRI), 61, Cheomdan-ro, Daegu 41062, Republic of Korea; ^3^Department of Brain and Cognitive Sciences, Daegu Gyeongbuk Institute of Science & Technology, Daegu 42988, Republic of Korea

**Running title:** The effect of chronic oral administration of ibrutinib on AD pathology

**Key words**: ibrutinib, Aβ, tau, cognitive function, neuroinflammation

*** Corresponding author:**

**Hyang-Sook Hoe, PhD**: Department of Neural Development and Disease, Korea Brain Research Institute (KBRI), 61, Cheomdan-ro, Daegu, Republic of Korea, Tel: 82-53-980-8310, Fax: 85-53-980-8309, E-mail: sookhoe72@kbri.re.kr

**Hyun-ju Lee, PhD**: Department of Neural Development and Disease, Korea Brain Research Institute (KBRI), 61, Cheomdan-ro, Daegu, Republic of Korea, Tel: 82-53-980-8313, Fax: 85-53-980-8309, E-mail: [hjlee@kbri.re.kr](mailto:hjlee@kbri.re.kr)

Hyun-ju Lee: hjlee@kbri.re.kr

Sora Kang: [ksr8947@kbri.re.kr](mailto:ksr8947@kbri.re.kr)

Yoo Joo Jeong: yoojoo930@kbri.re.kr

Jin-Hee Park: [mingmeng1005@kbri.re.kr](mailto:mingmeng1005@kbri.re.kr)

Jeong-Woo Hwang: [jmseil42@kbri.re.kr](mailto:jmseil42@kbri.re.kr)

Chan-Hu Gu: a422a422@kbri.re.kr

Tae-Mi Jung: taemi1514@kbri.re.kr

Seokjun Oh: dk05056@kbri.re.kr

Ji-Yeong Jang: [jangjy@kbri.re.kr](mailto:jangjy@kbri.re.kr)

Hyang-Sook Hoe: [sookhoe72@kbri.re.kr](mailto:sookhoe72@kbri.re.kr)

**
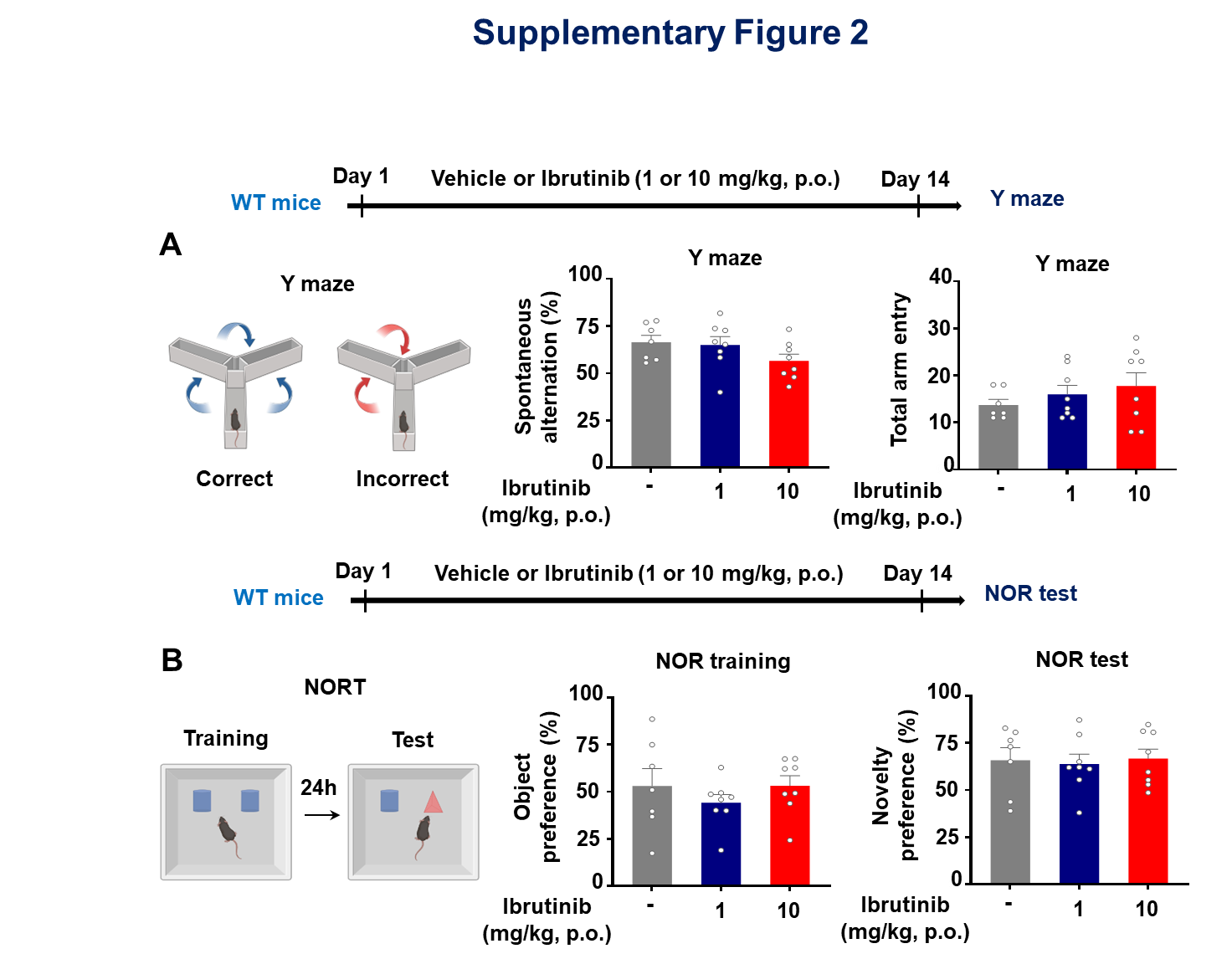
Supplementary Fig.1. Daily oral administration of ibrutinib for 2 weeks does not affect short-term and recognition memory in WT mice.** WT mice (6 weeks old, male) were administered vehicle (5% DMSO + 30% PEG + 5% Tween 80 + 60% ddH_2_O) or ibrutinib (1 or 10 mg/kg, p.o.) by oral gavage daily for 2 weeks. (A-B) Short-term and recognition memory were evaluated by Y maze and novel object recognition (NOR) test, respectively (n = 7-8 mice/group).

**
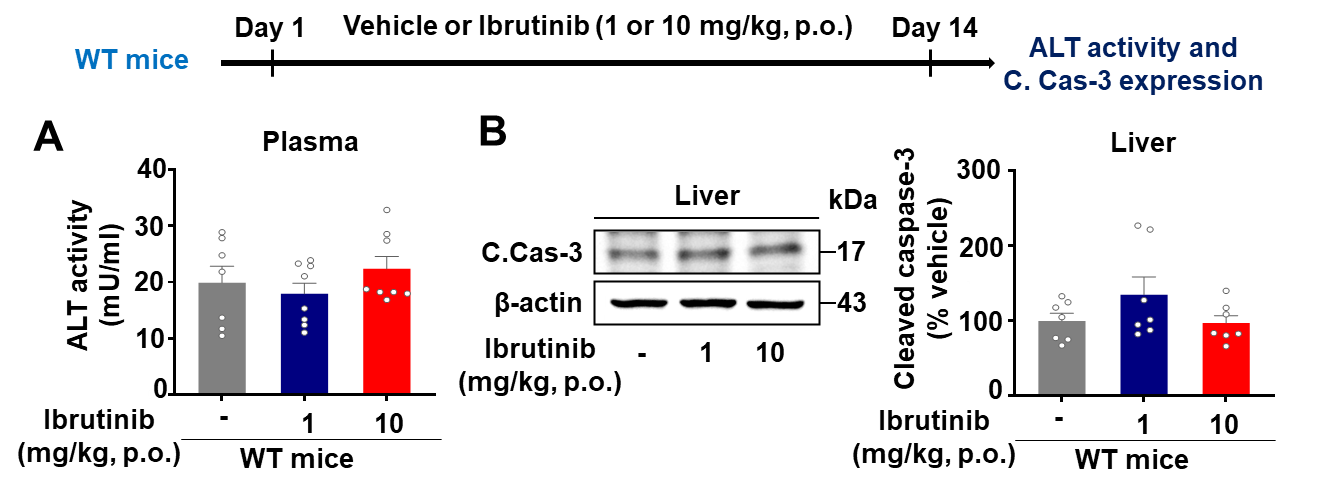
**

**Supplementary Fig.2. Daily oral administration of ibrutinib for 2 weeks did not alter plasma ALT activity and cleaved caspase-3 levels in WT mice.** WT mice (6 weeks old, male) were administered vehicle (5% DMSO + 30% PEG + 5% Tween 80 + 60% ddH_2_O) or ibrutinib (1 or 10 mg/kg, p.o.) by oral gavage daily for 2 weeks. (A-B) The hepatotoxicity of ibrutinib (1 or 10 mg/kg, p.o., daily for 2 weeks) was evaluated by measuring plasma ALT activity (n =7-8 mice/group) and by western blot analysis of cleaved caspase-3 in liver lysates (n = 7 mice/group).

**
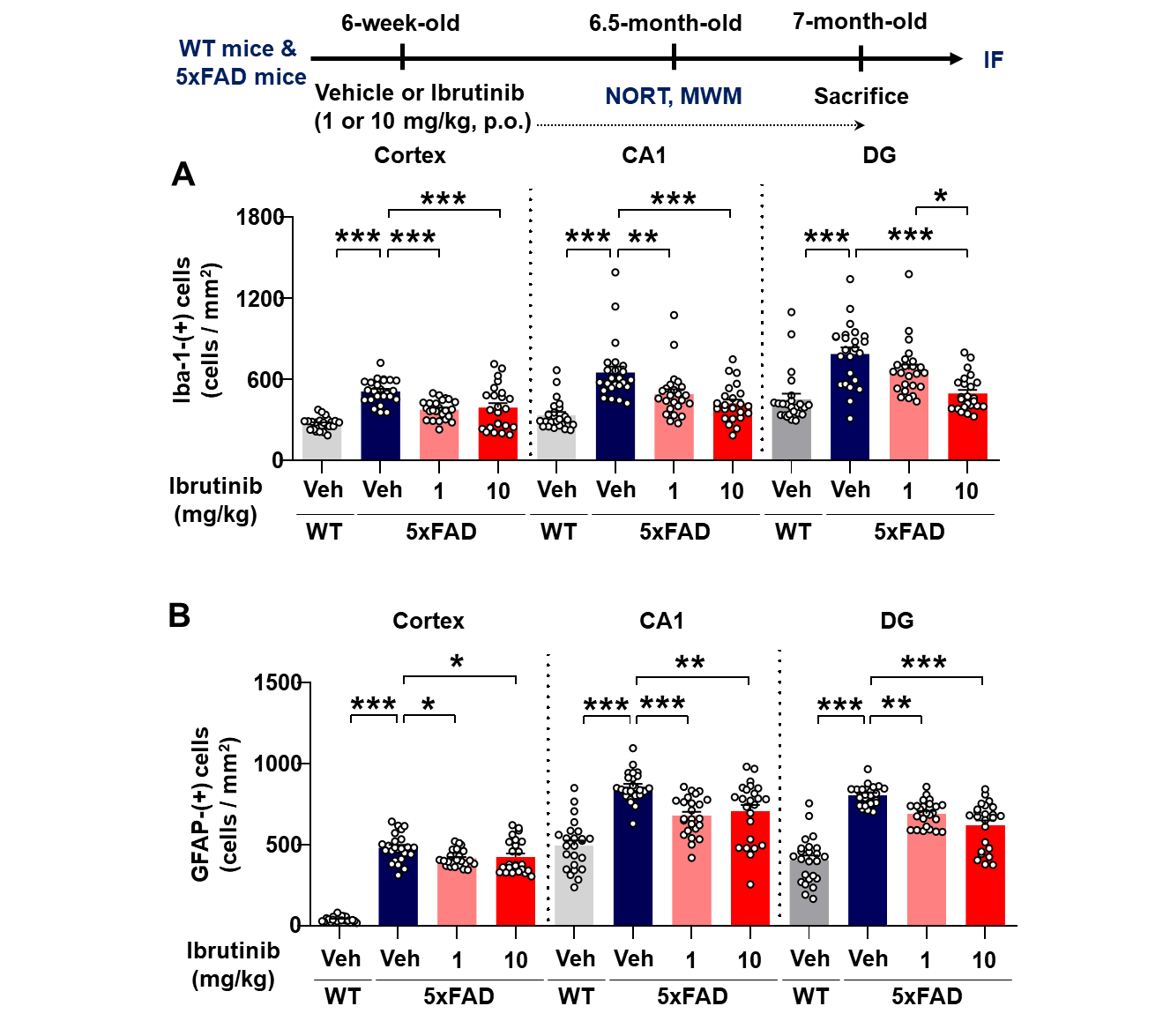
Supplementary Fig. 3. Chronic oral administration of ibrutinib decreases microglial and astroglial migration in 5xFAD mice.** WT mice and 5xFAD mice (6 weeks old, male) were administered vehicle (5% DMSO + 30% PEG + 5% Tween 80 + 60% ddH_2_O) or ibrutinib (1 or 10 mg/kg, p.o.) by oral gavage daily for 5 months. (A-B) Quantification of IF staining of mouse brain slices with anti-Iba-1 (A) or anti-GFAP (B) (n = 24 brain slices from 6 mice/group). **p* < 0.05, ***p* < 0.01, and ****p* < 0.001.

**Supplementary Materials and Methods**

**5xFAD mice and wild-type mice**

All experimental procedures were approved by the institutional biosafety committee (IBC) and were performed in accordance with approved animal protocols and guidelines of the Korea Brain Research Institute (KBRI, approval nos. IACUC-19-00049 and IACUC-23-00020). Six-week-old 5xFAD hemizygous (Jackson Laboratory, stock # 34848-JAX; B6Cg-Tg APPSwFlLon, PSEN1*M146L*L286V6799Vas/Mmjax) and C57BL6/N male mice [wild-type (WT); Orient-Bio Company, Gyeonggi-do, Korea] were used for *in vivo* experiments and randomly assigned to experimental groups. The mice were maintained in a pathogen-free facility with a 12-h photoperiod in cages housing 3–4 mice each and had access to food and water *ad libitum.*

**Ibrutinib administration**

Ibrutinib (S2680, Selleck Chemicals, Houston, TX) was dissolved in vehicle (5% DMSO, 30% PEG, 5% Tween 80, 60% ddH_2_O) at a dose of 1 or 10 mg/kg.

**Behavioral tests**

To examine whether chronic oral administration of ibrutinib modulates cognitive function and AD pathology, six-week-old male 5xFAD mice were administered ibrutinib (1 or 10 mg/kg, p.o.) or vehicle (5% DMSO, 30% PEG, 5% Tween 80, 60% ddH_2_O) and WT mice were treated with vehicle (5% DMSO, 30% PEG, 5% Tween 80, 60% ddH_2_O) daily for 5 months. After chronic oral administration of ibrutinib (1 or 10 mg/kg, p.o., daily for 5 months), the novel object recognition (NOR) test and Morris water maze (MWM) test were performed to analyze the effects of ibrutinib on long-term recognition memory and hippocampus-dependent spatial memory, respectively, as previously described [1; 2].

To determine the effects of ibrutinib on short-term memory, recognition memory, and ALT activity in WT mice, WT mice were administered with ibrutinib (1 or 10 mg/kg, p.o.) or vehicle (5% DMSO, 30% PEG, 5% Tween 80, 60% ddH_2_O) daily for 2 weeks. After the 2 weeks of administration, Y maze and NOR test were performed as previously described [1].

**Immunofluorescence staining**

After the behavioral tests, the effect of chronic oral administration of ibrutinib on AD pathology in 5xFAD mice and WT mice was examined by performing immunofluorescence (IF) staining of brain slices as previously described [1; 2]. Briefly, cryo-sectioned mice brain slices were immunostained for 24–72 h at 4 °C with anti-β-Amyloid,17-24 antibody, anti-Tau^Ser202/Thr205^, anti-Tau^Thr212/Ser214^, anti-p-GSK-3α/β, anti-Iba-1, anti-GFAP, or anti-NLRP3 antibodies. Detailed information on the antibodies is provided in Supplementary Table 1. Images of the sections were acquired by fluorescence microscopy (DMi8, Leica Microsystems, Wetzlar, Germany) and analyzed by the software ImageJ (version 1.53a, National Institutes of Health, Bethesda, MD, USA).

**Alanine aminotransferase activity assay**

Serum alanine aminotransferase (ALT) activity, an indicator of liver injury, was analyzed to determine whether chronic administration of ibrutinib by oral gavage induces hepatotoxicity. In addition, we investigated whether oral administration of ibrutinib daily for 2 weeks modulates liver toxicity in WT mice, the cardiac plasma ALT activity was measured according to the manufacturer’s instructions (Cat no. MAK052, Sigma-Aldrich). Briefly, the plasma and the master reaction mixture containing ALT assay buffer, fluorescent peroxidase substrate, ALT enzyme mix, and ALT substrate was loaded into a 96-well plate. After 2 min, the initial intensity (γ_ex_ = 535 nm/γ_em_ = 587 nm) was measured. After incubation at 37 °C for 15 min (T_final_), and the final fluorescence intensity was measured. ALT activity was calculated using the following equation: ALT activity = [(nmol of pyruvate generated between T_initial_ and T_final_) * sample dilution factor]/[(T_final_ -T_initial_) * sample volume].

**Western blot**

The potential hepatoxicity of chronic oral administration of ibrutinib was evaluated by western blot analysis of cleaved caspase-3 protein levels in liver lysates of 5xFAD mice and WT mice. After the behavioral tests, the liver was dissected and homogenized in RIPA lysis buffer (Merck Millipore, Billerica, MA, USA) containing 1% protease and phosphatase inhibitor cocktail (Thermo Scientific, Waltham, MA, USA). Then, the proteins in the supernatant of liver lysate were separated by electrophoresis, and electrotransferred to a PVDF membrane (Millipore, Bedford, MA, USA). The membrane was blocked with 5% skim milk in TBST and incubated overnight with anti-cleaved caspase 3 antibody or anti-β-actin antibody at 4 °C. Next, the membrane was incubated with the horseradish peroxidase-conjugated secondary antibody for 1 h at RT. Finally, ECL solution (ATTO, Tokyo, Japan) was added for detection, and images were acquired and analyzed with the software Fusion Capt Advance (Vilber Lourmat, Eberhardzell, Germany). Detailed information on the antibodies is provided in Supplementary Table 1.

**Real-time quantitative PCR**

The effect of ibrutinib on NLRP3 mRNA levels in WT mice and 5xFAD mice was assessed by real-time quantitative PCR. For this experiment, six-week-old male 5xFAD mice were administered ibrutinib (1 or 10 mg/kg, p.o.) or vehicle (5% DMSO, 30% PEG, 5% Tween 80, 60% ddH_2_O) and WT mice were treated with vehicle (5% DMSO, 30% PEG, 5% Tween 80, 60% ddH_2_O) daily for 5 months. After the behavioral tests, the cortex and hippocampus were dissected and total RNA was extracted, reverse transcribed to cDNA, and used in real-time quantitative PCR (qPCR) as described by [1]. The value for GAPDH was used to normalize cycle threshold (Ct) values, and the fold change relative to the control (wild type treated with vehicle) was calculated. Detailed information on the primer sequence is provided in Supplementary Table 2.

**Statistical analysis**

Graphs were generated in GraphPad Prism 7 (GraphPad Software, San Diego, CA, USA), which was also used for statistical analysis. The results are presented as individual data and means ± SEMs. One-way analysis of variance (ANOVA) with Tukey’s or Dunnett's T3 multiple-comparisons test was used for multiple comparisons. Asterisks indicate significance: * *p* < 0.05, ***p* < 0.01, and *** *p* < 0.001. Detailed statistical results are provided in Supplementary Table 3.

**Supplementary Table 1.** List of antibodies used in this study

| Primary antibodies | | | | | | |
| --- | --- | --- | --- | --- | --- | --- |
| **Immunogen** | | **Host species** | **Dilution** | **Manufacturer** | **Catalog no.** | **Application** |
| β-Amyloid,17-24 | Mouse | | 1:500 | BioLegend | 800704 | IF |
| Tau^Ser202/Thr205^ | Mouse | | 1:100 | Invitrogen | MN1020 | IF |
| Tau^Thr212/Ser214^ | Mouse | | 1:200 | Invitrogen | MN1060 | IF |
| p-GSK-3α/β | Rabbit | | 1:200 | Abcam | AB75745 | IF |
| Iba-1 | Rabbit | | 1:500 | Wako | 019-19741 | IF |
| GFAP | Rat | | 1:500 | Invitrogen | 13-0300 | IF |
| NLRP3 | Rabbit | | 1:200 | Novus | NBP2-12446 | IF |
| Cleaved caspase-3 | Rabbit | | 1:500 | Cell Signaling | 9664 | WB |
| β-actin | Mouse | | 1:1000 | Santa Cruz | SC-47778 | WB |
| **Secondary antibodies** | | | | | | |
| **Antibody** | | | **Dilution** | **Manufacturer** | **Catalog no.** | **Application** |
| Goat anti-rabbit IgG, Alexa Fluor™ 594 | | | 1:200 | Invitrogen | A11012 | IF |
| Goat anti-mouse IgG, Alexa Fluor™ 488 | | | 1:200 | Invitrogen | A11001 | IF |
| Goat anti-rat IgG, Alexa Fluor™ 488 | | | 1:200 | Invitrogen | A11006 | IF |
| Goat anti-rabbit, HRP | | | 1:5000 | Enzo | ADI-SAB-300-J | WB |
| Goat anti-mouse, HRP | | | 1:5000 | Enzo | ADI-SAB-100-J | WB |

Supplementary Table 2. Primer sequences for real-time qPCR

| Gene |  | Sequence |
| --- | --- | --- |
| NLRP3 | Forward | 5’-TCC ACA ATT CTG ACC CAC AA-3’ |
|  | Reverse | 5’-ACC TCA CAG AGG GTC ACC AC-3’ |
| GAPDH | Forward | 5’- TGT GTC CGT CGT GGA TCT GA-3’ |
|  | Reverse | 5’-CCT GCTTCA CCA CCT TCT TGA-3’ |

**Supplementary Table 3.** Statistical analysis results of the *in vivo* experiments.

| **Figure 1A. Novel Object Recognition Training** |
| --- |
| \| Table Analyzed \| NOR training \| \| --- \| --- \| \| Data sets analyzed \| A-D \| \|  \|  \| \| ANOVA summary \|  \| \| F \| 0.9090 \| \| P value \| 0.4453 \| \| P value summary \| ns \| \| Significant diff. among means (P < 0.05)? \| No \| \| R square \| 0.06382 \|  \| Number of families \| 1 \|  \|  \|  \|  \|  \| \| --- \| --- \| --- \| --- \| --- \| --- \| --- \| \| Number of comparisons per family \| 6 \|  \|  \|  \|  \|  \| \| Alpha \| 0.05 \|  \|  \|  \|  \|  \| \|  \|  \|  \|  \|  \|  \|  \| \| Tukey's multiple comparisons test \| Mean Diff. \| 95.00% CI of diff. \| Significant? \| Summary \| Adjusted P Value \|  \| \| Veh vs. Veh \| -4.824 \| -24.20 to 14.56 \| No \| ns \| 0.9088 \| A-B \| \| Veh vs. Ibr-1 \| -4.080 \| -23.05 to 14.89 \| No \| ns \| 0.9386 \| A-C \| \| Veh vs. Ibr-10 \| 6.062 \| -13.80 to 25.92 \| No \| ns \| 0.8456 \| A-D \| \| Veh vs. Ibr-1 \| 0.7438 \| -18.23 to 19.72 \| No \| ns \| 0.9996 \| B-C \| \| Veh vs. Ibr-10 \| 10.89 \| -8.974 to 30.75 \| No \| ns \| 0.4652 \| B-D \| \| Ibr-1 vs. Ibr-10 \| 10.14 \| -9.320 to 29.60 \| No \| ns \| 0.5087 \| C-D \| |
| **Figure 1A. Novel Object Recognition Test** |
| \| Table Analyzed \| NORT \| \| --- \| --- \| \| Data sets analyzed \| A-D \| \|  \|  \| \| ANOVA summary \|  \| \| F \| 6.912 \| \| P value \| 0.0007 \| \| P value summary \| *** \| \| Significant diff. among means (P < 0.05)? \| Yes \| \| R square \| 0.3414 \|  \| Number of families \| 1 \|  \|  \|  \|  \|  \|  \|  \|  \|  \|  \|  \|  \| \| --- \| --- \| --- \| --- \| --- \| --- \| --- \| --- \| --- \| --- \| --- \| --- \| --- \| --- \| \| Number of comparisons per family \| 6 \|  \|  \|  \|  \|  \|  \|  \|  \|  \|  \|  \|  \| \| Alpha \| 0.05 \|  \|  \|  \|  \|  \|  \|  \|  \|  \|  \|  \|  \|  \| Number of families \| 1 \|  \|  \|  \|  \|  \| \| --- \| --- \| --- \| --- \| --- \| --- \| --- \| \| Number of comparisons per family \| 6 \|  \|  \|  \|  \|  \| \| Alpha \| 0.05 \|  \|  \|  \|  \|  \| \|  \|  \|  \|  \|  \|  \|  \| \| Tukey's multiple comparisons test \| Mean Diff. \| 95.00% CI of diff. \| Significant? \| Summary \| Adjusted P Value \|  \| \| Veh vs. Veh \| 22.72 \| 9.009 to 36.43 \| Yes \| *** \| 0.0004 \| A-B \| \| Veh vs. Ibr-1 \| 13.74 \| 0.3155 to 27.16 \| Yes \| * \| 0.0431 \| A-C \| \| Veh vs. Ibr-10 \| 8.577 \| -5.472 to 22.63 \| No \| ns \| 0.3704 \| A-D \| \| Veh vs. Ibr-1 \| -8.982 \| -22.40 to 4.441 \| No \| ns \| 0.2913 \| B-C \| \| Veh vs. Ibr-10 \| -14.14 \| -28.19 to -0.09268 \| Yes \| * \| 0.0480 \| B-D \| \| Ibr-1 vs. Ibr-10 \| -5.161 \| -18.93 to 8.608 \| No \| ns \| 0.7476 \| C-D \| |
| **Figure 1B. Morris Water Maze- Acquisition test (Day 1~ Day 4)** |
| \| Table Analyzed \| acquisition \|  \|  \|  \| \| --- \| --- \| --- \| --- \| --- \| \|  \|  \|  \|  \|  \| \| Two-way ANOVA \| Ordinary \|  \|  \|  \| \| Alpha \| 0.05 \|  \|  \|  \| \|  \|  \|  \|  \|  \| \| Source of Variation \| % of total variation \| P value \| P value summary \| Significant? \| \| Interaction \| 4.707 \| 0.2608 \| ns \| No \| \| Row Factor \| 7.027 \| 0.0010 \| ** \| Yes \| \| Column Factor \| 13.88 \| <0.0001 \| **** \| Yes \|  \| Within each row, compare columns (simple effects within rows) \|  \|  \|  \|  \|  \| \| --- \| --- \| --- \| --- \| --- \| --- \| \|  \|  \|  \|  \|  \|  \| \| Number of families \| 4 \|  \|  \|  \|  \| \| Number of comparisons per family \| 6 \|  \|  \|  \|  \| \| Alpha \| 0.05 \|  \|  \|  \|  \| \|  \|  \|  \|  \|  \|  \| \| Tukey's multiple comparisons test \| Mean Diff. \| 95.00% CI of diff. \| Significant? \| Summary \| Adjusted P Value \| \|  \|  \|  \|  \|  \|  \| \| Day 1 \|  \|  \|  \|  \|  \| \| WT+Veh vs. 5xFAD+Veh \| -9.181 \| -21.72 to 3.356 \| No \| ns \| 0.2321 \| \| WT+Veh vs. 5xFAD+Ibu_1mpk \| -3.139 \| -15.43 to 9.154 \| No \| ns \| 0.9111 \| \| WT+Veh vs. 5xFAD+Ibu_10mpk \| -9.283 \| -21.82 to 3.253 \| No \| ns \| 0.2232 \| \| 5xFAD+Veh vs. 5xFAD+Ibu_1mpk \| 6.042 \| -6.251 to 18.34 \| No \| ns \| 0.5803 \| \| 5xFAD+Veh vs. 5xFAD+Ibu_10mpk \| -0.1022 \| -12.64 to 12.43 \| No \| ns \| >0.9999 \| \| 5xFAD+Ibu_1mpk vs. 5xFAD+Ibu_10mpk \| -6.144 \| -18.44 to 6.149 \| No \| ns \| 0.5666 \| \|  \|  \|  \|  \|  \|  \| \| Day 2 \|  \|  \|  \|  \|  \| \| WT+Veh vs. 5xFAD+Veh \| -13.07 \| -25.61 to -0.5357 \| Yes \| * \| 0.0373 \| \| WT+Veh vs. 5xFAD+Ibu_1mpk \| -4.528 \| -16.82 to 7.765 \| No \| ns \| 0.7749 \| \| WT+Veh vs. 5xFAD+Ibu_10mpk \| -15.40 \| -27.94 to -2.862 \| Yes \| ** \| 0.0092 \| \| 5xFAD+Veh vs. 5xFAD+Ibu_1mpk \| 8.544 \| -3.749 to 20.84 \| No \| ns \| 0.2756 \| \| 5xFAD+Veh vs. 5xFAD+Ibu_10mpk \| -2.327 \| -14.86 to 10.21 \| No \| ns \| 0.9632 \| \| 5xFAD+Ibu_1mpk vs. 5xFAD+Ibu_10mpk \| -10.87 \| -23.16 to 1.422 \| No \| ns \| 0.1034 \| \|  \|  \|  \|  \|  \|  \| \| Day 3 \|  \|  \|  \|  \|  \| \| WT+Veh vs. 5xFAD+Veh \| -13.22 \| -25.76 to -0.6849 \| Yes \| * \| 0.0344 \| \| WT+Veh vs. 5xFAD+Ibu_1mpk \| -13.38 \| -25.67 to -1.085 \| Yes \| * \| 0.0270 \| \| WT+Veh vs. 5xFAD+Ibu_10mpk \| -7.376 \| -19.91 to 5.161 \| No \| ns \| 0.4243 \| \| 5xFAD+Veh vs. 5xFAD+Ibu_1mpk \| -0.1570 \| -12.45 to 12.14 \| No \| ns \| >0.9999 \| \| 5xFAD+Veh vs. 5xFAD+Ibu_10mpk \| 5.846 \| -6.691 to 18.38 \| No \| ns \| 0.6218 \| \| 5xFAD+Ibu_1mpk vs. 5xFAD+Ibu_10mpk \| 6.003 \| -6.290 to 18.30 \| No \| ns \| 0.5856 \| \|  \|  \|  \|  \|  \|  \| \| Day 4 \|  \|  \|  \|  \|  \| \| WT+Veh vs. 5xFAD+Veh \| -17.31 \| -29.85 to -4.776 \| Yes \| ** \| 0.0025 \| \| WT+Veh vs. 5xFAD+Ibu_1mpk \| -15.13 \| -27.42 to -2.838 \| Yes \| ** \| 0.0090 \| \| WT+Veh vs. 5xFAD+Ibu_10mpk \| -9.519 \| -22.06 to 3.018 \| No \| ns \| 0.2036 \| \| 5xFAD+Veh vs. 5xFAD+Ibu_1mpk \| 2.182 \| -10.11 to 14.47 \| No \| ns \| 0.9676 \| \| 5xFAD+Veh vs. 5xFAD+Ibu_10mpk \| 7.794 \| -4.743 to 20.33 \| No \| ns \| 0.3744 \| \| 5xFAD+Ibu_1mpk vs. 5xFAD+Ibu_10mpk \| 5.612 \| -6.681 to 17.91 \| No \| ns \| 0.6378 \| |
| **Figure 1C. Morris Water Maze- Acquisition test (Day 1 vs. Day 4)** |
| \| Table Analyzed \| Day 1 vs Day 4 \|  \|  \|  \| \| --- \| --- \| --- \| --- \| --- \| \|  \|  \|  \|  \|  \| \| Two-way RM ANOVA \| Matching: Across row \|  \|  \|  \| \| Assume sphericity? \| Yes \|  \|  \|  \| \| Alpha \| 0.05 \|  \|  \|  \| \|  \|  \|  \|  \|  \| \| Source of Variation \| % of total variation \| P value \| P value summary \| Significant? \| \| Interaction \| 3.740 \| 0.1629 \| ns \| No \| \| Row Factor \| 12.87 \| 0.0049 \| ** \| Yes \| \| Time \| 13.11 \| <0.0001 \| **** \| Yes \| \| Subject \| 39.27 \| 0.2269 \| ns \| No \|  \| Compare each cell mean with the other cell mean in that row \|  \|  \|  \|  \|  \| \| --- \| --- \| --- \| --- \| --- \| --- \| \|  \|  \|  \|  \|  \|  \| \| Number of families \| 1 \|  \|  \|  \|  \| \| Number of comparisons per family \| 4 \|  \|  \|  \|  \| \| Alpha \| 0.05 \|  \|  \|  \|  \| \|  \|  \|  \|  \|  \|  \| \| Bonferroni's multiple comparisons test \| Predicted (LS) mean diff. \| 95.00% CI of diff. \| Significant? \| Summary \| Adjusted P Value \| \|  \|  \|  \|  \|  \|  \| \| Day 1 vs Day 4 \|  \|  \|  \|  \|  \| \| Veh \| 14.83 \| 3.031 to 26.62 \| Yes \| ** \| 0.0082 \| \| Veh \| 6.695 \| -5.101 to 18.49 \| No \| ns \| 0.5867 \| \| Ibr-1 \| 2.834 \| -8.498 to 14.17 \| No \| ns \| >0.9999 \| \| Ibr-10 \| 14.59 \| 2.796 to 26.39 \| Yes \| ** \| 0.0096 \| |
| **Figure 1D. ALT activity in serum** |
| \| Table Analyzed \| ALT activity \| \| --- \| --- \| \| Data sets analyzed \| A-D \| \|  \|  \| \| ANOVA summary \|  \| \| F \| 0.6653 \| \| P value \| 0.5788 \| \| P value summary \| ns \| \| Significant diff. among means (P < 0.05)? \| No \| \| R square \| 0.05253 \|  \| Number of families \| 1 \|  \|  \|  \|  \|  \| \| --- \| --- \| --- \| --- \| --- \| --- \| --- \| \| Number of comparisons per family \| 6 \|  \|  \|  \|  \|  \| \| Alpha \| 0.05 \|  \|  \|  \|  \|  \| \|  \|  \|  \|  \|  \|  \|  \| \| Tukey's multiple comparisons test \| Mean Diff. \| 95.00% CI of diff. \| Significant? \| Summary \| Adjusted P Value \|  \| \| Veh vs. Veh \| -3.894 \| -17.07 to 9.281 \| No \| ns \| 0.8558 \| A-B \| \| Veh vs. Ibr-1 \| 2.780 \| -10.39 to 15.95 \| No \| ns \| 0.9409 \| A-C \| \| Veh vs. Ibr-10 \| 0.9983 \| -12.18 to 14.17 \| No \| ns \| 0.9969 \| A-D \| \| Veh vs. Ibr-1 \| 6.674 \| -6.501 to 19.85 \| No \| ns \| 0.5294 \| B-C \| \| Veh vs. Ibr-10 \| 4.892 \| -8.283 to 18.07 \| No \| ns \| 0.7503 \| B-D \| \| Ibr-1 vs. Ibr-10 \| -1.782 \| -14.96 to 11.39 \| No \| ns \| 0.9832 \| C-D \| |
| **Figure 1F. Cleaved caspase-3 levels in liver** |
| \| Table Analyzed \| C.Cas-3 in liver \| \| --- \| --- \| \| Data sets analyzed \| A-D \| \|  \|  \| \| ANOVA summary \|  \| \| F \| 0.4453 \| \| P value \| 0.7233 \| \| P value summary \| ns \| \| Significant diff. among means (P < 0.05)? \| No \| \| R square \| 0.06262 \|  \| Number of families \| 1 \|  \|  \|  \|  \|  \| \| --- \| --- \| --- \| --- \| --- \| --- \| --- \| \| Number of comparisons per family \| 6 \|  \|  \|  \|  \|  \| \| Alpha \| 0.05 \|  \|  \|  \|  \|  \| \|  \|  \|  \|  \|  \|  \|  \| \| Tukey's multiple comparisons test \| Mean Diff. \| 95.00% CI of diff. \| Significant? \| Summary \| Adjusted P Value \|  \| \| Veh vs. Veh \| -33.60 \| -115.0 to 47.77 \| No \| ns \| 0.6604 \| A-B \| \| Veh vs. Ibr-1 \| -16.58 \| -97.95 to 64.79 \| No \| ns \| 0.9398 \| A-C \| \| Veh vs. Ibr-10 \| -16.68 \| -98.05 to 64.70 \| No \| ns \| 0.9388 \| A-D \| \| Veh vs. Ibr-1 \| 17.02 \| -64.35 to 98.40 \| No \| ns \| 0.9353 \| B-C \| \| Veh vs. Ibr-10 \| 16.93 \| -64.45 to 98.30 \| No \| ns \| 0.9363 \| B-D \| \| Ibr-1 vs. Ibr-10 \| -0.09713 \| -81.47 to 81.28 \| No \| ns \| >0.9999 \| C-D \| |
| **Figure 1H. Aβ plaque numbers in Cortex** |
| \| Table Analyzed \| 4G8 in Cortex \| \| --- \| --- \| \| Data sets analyzed \| A-D \| \|  \|  \| \| ANOVA summary \|  \| \| F \| 25.12 \| \| P value \| <0.0001 \| \| P value summary \| **** \| \| Significant diff. among means (P < 0.05)? \| Yes \| \| R square \| 0.4503 \|  \| Number of families \| 1 \|  \|  \|  \|  \|  \| \| --- \| --- \| --- \| --- \| --- \| --- \| --- \| \| Number of comparisons per family \| 6 \|  \|  \|  \|  \|  \| \| Alpha \| 0.05 \|  \|  \|  \|  \|  \| \|  \|  \|  \|  \|  \|  \|  \| \| Tukey's multiple comparisons test \| Mean Diff. \| 95.00% CI of diff. \| Significant? \| Summary \| Adjusted P Value \|  \| \| Veh vs. Veh \| -14.84 \| -19.65 to -10.04 \| Yes \| **** \| <0.0001 \| A-B \| \| Veh vs. Ibr-1 \| -10.38 \| -15.18 to -5.569 \| Yes \| **** \| <0.0001 \| A-C \| \| Veh vs. Ibr-10 \| -4.548 \| -9.354 to 0.2579 \| No \| ns \| 0.0704 \| A-D \| \| Veh vs. Ibr-1 \| 4.466 \| -0.3398 to 9.273 \| No \| ns \| 0.0782 \| B-C \| \| Veh vs. Ibr-10 \| 10.29 \| 5.488 to 15.10 \| Yes \| **** \| <0.0001 \| B-D \| \| Ibr-1 vs. Ibr-10 \| 5.827 \| 1.021 to 10.63 \| Yes \| * \| 0.0109 \| C-D \| |
| **Figure 1H. Aβ plaque numbers in subiculum** |
| \| Table Analyzed \| 4G8 in Subiculum \| \| --- \| --- \| \| Data sets analyzed \| A-D \| \|  \|  \| \| ANOVA summary \|  \| \| F \| 59.51 \| \| P value \| <0.0001 \| \| P value summary \| **** \| \| Significant diff. among means (P < 0.05)? \| Yes \| \| R square \| 0.6599 \|  \| Number of families \| 1 \|  \|  \|  \|  \|  \| \| --- \| --- \| --- \| --- \| --- \| --- \| --- \| \| Number of comparisons per family \| 6 \|  \|  \|  \|  \|  \| \| Alpha \| 0.05 \|  \|  \|  \|  \|  \| \|  \|  \|  \|  \|  \|  \|  \| \| Tukey's multiple comparisons test \| Mean Diff. \| 95.00% CI of diff. \| Significant? \| Summary \| Adjusted P Value \|  \| \| Veh vs. Veh \| -97.29 \| -116.6 to -78.01 \| Yes \| **** \| <0.0001 \| A-B \| \| Veh vs. Ibr-1 \| -61.86 \| -81.14 to -42.57 \| Yes \| **** \| <0.0001 \| A-C \| \| Veh vs. Ibr-10 \| -53.90 \| -73.19 to -34.62 \| Yes \| **** \| <0.0001 \| A-D \| \| Veh vs. Ibr-1 \| 35.44 \| 16.15 to 54.72 \| Yes \| **** \| <0.0001 \| B-C \| \| Veh vs. Ibr-10 \| 43.39 \| 24.10 to 62.68 \| Yes \| **** \| <0.0001 \| B-D \| \| Ibr-1 vs. Ibr-10 \| 7.952 \| -11.34 to 27.24 \| No \| ns \| 0.7033 \| C-D \| |
| **Figure 1H. Aβ plaque numbers in CA1** |
| \| Table Analyzed \| 4G8 in CA1 \| \| --- \| --- \| \| Data sets analyzed \| A-D \| \|  \|  \| \| ANOVA summary \|  \| \| F \| 26.57 \| \| P value \| <0.0001 \| \| P value summary \| **** \| \| Significant diff. among means (P < 0.05)? \| Yes \| \| R square \| 0.4642 \|  \| Number of families \| 1 \|  \|  \|  \|  \|  \| \| --- \| --- \| --- \| --- \| --- \| --- \| --- \| \| Number of comparisons per family \| 6 \|  \|  \|  \|  \|  \| \| Alpha \| 0.05 \|  \|  \|  \|  \|  \| \|  \|  \|  \|  \|  \|  \|  \| \| Tukey's multiple comparisons test \| Mean Diff. \| 95.00% CI of diff. \| Significant? \| Summary \| Adjusted P Value \|  \| \| Veh vs. Veh \| -27.98 \| -36.21 to -19.75 \| Yes \| **** \| <0.0001 \| A-B \| \| Veh vs. Ibr-1 \| -12.63 \| -20.86 to -4.401 \| Yes \| *** \| 0.0007 \| A-C \| \| Veh vs. Ibr-10 \| -12.10 \| -20.33 to -3.868 \| Yes \| ** \| 0.0012 \| A-D \| \| Veh vs. Ibr-1 \| 15.35 \| 7.121 to 23.58 \| Yes \| **** \| <0.0001 \| B-C \| \| Veh vs. Ibr-10 \| 15.88 \| 7.654 to 24.11 \| Yes \| **** \| <0.0001 \| B-D \| \| Ibr-1 vs. Ibr-10 \| 0.5330 \| -7.696 to 8.762 \| No \| ns \| 0.9983 \| C-D \| |
| **Figure 1H. Aβ plaque numbers in DG** |
| \| Table Analyzed \| 4G8 in DG \| \| --- \| --- \| \| Data sets analyzed \| A-D \| \|  \|  \| \| ANOVA summary \|  \| \| F \| 13.39 \| \| P value \| <0.0001 \| \| P value summary \| **** \| \| Significant diff. among means (P < 0.05)? \| Yes \| \| R square \| 0.3039 \|  \| Number of families \| 1 \|  \|  \|  \|  \|  \| \| --- \| --- \| --- \| --- \| --- \| --- \| --- \| \| Number of comparisons per family \| 6 \|  \|  \|  \|  \|  \| \| Alpha \| 0.05 \|  \|  \|  \|  \|  \| \|  \|  \|  \|  \|  \|  \|  \| \| Tukey's multiple comparisons test \| Mean Diff. \| 95.00% CI of diff. \| Significant? \| Summary \| Adjusted P Value \|  \| \| Veh vs. Veh \| -8.478 \| -12.27 to -4.685 \| Yes \| **** \| <0.0001 \| A-B \| \| Veh vs. Ibr-1 \| -7.294 \| -11.09 to -3.501 \| Yes \| **** \| <0.0001 \| A-C \| \| Veh vs. Ibr-10 \| -5.051 \| -8.844 to -1.258 \| Yes \| ** \| 0.0041 \| A-D \| \| Veh vs. Ibr-1 \| 1.184 \| -2.609 to 4.977 \| No \| ns \| 0.8464 \| B-C \| \| Veh vs. Ibr-10 \| 3.427 \| -0.3660 to 7.220 \| No \| ns \| 0.0914 \| B-D \| \| Ibr-1 vs. Ibr-10 \| 2.243 \| -1.550 to 6.036 \| No \| ns \| 0.4139 \| C-D \| |
| **Figure 1J. Tau^Ser202/Thr205^ fluorescence intensity in the cortex** |
| \| Table Analyzed \| AT8 in cortex \| \| --- \| --- \| \| Data sets analyzed \| A-D \| \|  \|  \| \| ANOVA summary \|  \| \| F \| 18.43 \| \| P value \| <0.0001 \| \| P value summary \| **** \| \| Significant diff. among means (P < 0.05)? \| Yes \| \| R square \| 0.3832 \|  \| Number of families \| 1 \|  \|  \|  \|  \|  \| \| --- \| --- \| --- \| --- \| --- \| --- \| --- \| \| Number of comparisons per family \| 6 \|  \|  \|  \|  \|  \| \| Alpha \| 0.05 \|  \|  \|  \|  \|  \| \|  \|  \|  \|  \|  \|  \|  \| \| Tukey's multiple comparisons test \| Mean Diff. \| 95.00% CI of diff. \| Significant? \| Summary \| Adjusted P Value \|  \| \| Veh vs. Veh \| -108.3 \| -146.9 to -69.79 \| Yes \| **** \| <0.0001 \| A-B \| \| Veh vs. Ibr-1 \| -67.51 \| -106.1 to -28.96 \| Yes \| **** \| <0.0001 \| A-C \| \| Veh vs. Ibr-10 \| -61.13 \| -101.0 to -21.24 \| Yes \| *** \| 0.0007 \| A-D \| \| Veh vs. Ibr-1 \| 40.82 \| 2.277 to 79.37 \| Yes \| * \| 0.0336 \| B-C \| \| Veh vs. Ibr-10 \| 47.20 \| 7.300 to 87.09 \| Yes \| * \| 0.0137 \| B-D \| \| Ibr-1 vs. Ibr-10 \| 6.376 \| -33.52 to 46.27 \| No \| ns \| 0.9752 \| C-D \| |
| **Figure 1J. Tau^Ser202/Thr205^ fluorescence intensity in the hippocampal CA1** |
| \| Table Analyzed \| AT8-CA1 \| \| --- \| --- \| \| Data sets analyzed \| A-D \| \|  \|  \| \| ANOVA summary \|  \| \| F \| 16.72 \| \| P value \| <0.0001 \| \| P value summary \| **** \| \| Significant diff. among means (P < 0.05)? \| Yes \| \| R square \| 0.3529 \|  \| Number of families \| 1 \|  \|  \|  \|  \|  \| \| --- \| --- \| --- \| --- \| --- \| --- \| --- \| \| Number of comparisons per family \| 6 \|  \|  \|  \|  \|  \| \| Alpha \| 0.05 \|  \|  \|  \|  \|  \| \|  \|  \|  \|  \|  \|  \|  \| \| Tukey's multiple comparisons test \| Mean Diff. \| 95.00% CI of diff. \| Significant? \| Summary \| Adjusted P Value \|  \| \| Veh vs. Veh \| -75.33 \| -103.8 to -46.83 \| Yes \| **** \| <0.0001 \| A-B \| \| Veh vs. Ibr-1 \| -37.17 \| -65.67 to -8.672 \| Yes \| ** \| 0.0052 \| A-C \| \| Veh vs. Ibr-10 \| -51.06 \| -79.56 to -22.56 \| Yes \| **** \| <0.0001 \| A-D \| \| Veh vs. Ibr-1 \| 38.16 \| 9.662 to 66.66 \| Yes \| ** \| 0.0039 \| B-C \| \| Veh vs. Ibr-10 \| 24.27 \| -4.230 to 52.77 \| No \| ns \| 0.1233 \| B-D \| \| Ibr-1 vs. Ibr-10 \| -13.89 \| -42.39 to 14.61 \| No \| ns \| 0.5808 \| C-D \| |
| **Figure 1L. Tau^Thr212/Ser214^ fluorescence intensity in the Cortex** |
| \| Table Analyzed \| AT100 in cortex \| \| --- \| --- \| \| Data sets analyzed \| A-D \| \|  \|  \| \| ANOVA summary \|  \| \| F \| 3.786 \| \| P value \| 0.0131 \| \| P value summary \| * \| \| Significant diff. among means (P < 0.05)? \| Yes \| \| R square \| 0.1099 \|  \| Number of families \| 1 \|  \|  \|  \|  \|  \| \| --- \| --- \| --- \| --- \| --- \| --- \| --- \| \| Number of comparisons per family \| 6 \|  \|  \|  \|  \|  \| \| Alpha \| 0.05 \|  \|  \|  \|  \|  \| \|  \|  \|  \|  \|  \|  \|  \| \| Tukey's multiple comparisons test \| Mean Diff. \| 95.00% CI of diff. \| Significant? \| Summary \| Adjusted P Value \|  \| \| Veh vs. Veh \| -37.03 \| -65.98 to -8.077 \| Yes \| ** \| 0.0064 \| A-B \| \| Veh vs. Ibr-1 \| -16.36 \| -45.31 to 12.59 \| No \| ns \| 0.4546 \| A-C \| \| Veh vs. Ibr-10 \| -14.82 \| -43.77 to 14.13 \| No \| ns \| 0.5404 \| A-D \| \| Veh vs. Ibr-1 \| 20.67 \| -8.281 to 49.62 \| No \| ns \| 0.2489 \| B-C \| \| Veh vs. Ibr-10 \| 22.21 \| -6.744 to 51.16 \| No \| ns \| 0.1929 \| B-D \| \| Ibr-1 vs. Ibr-10 \| 1.537 \| -27.42 to 30.49 \| No \| ns \| 0.9990 \| C-D \| |
| **Figure 1L. Tau^Thr212/Ser214^ fluorescence intensity in the hippocampal CA1** |
| \| Table Analyzed \| AT100 in CA1 \| \| --- \| --- \| \| Data sets analyzed \| A-D \| \|  \|  \| \| ANOVA summary \|  \| \| F \| 7.782 \| \| P value \| 0.0001 \| \| P value summary \| *** \| \| Significant diff. among means (P < 0.05)? \| Yes \| \| R square \| 0.2024 \|  \| Number of families \| 1 \|  \|  \|  \|  \|  \| \| --- \| --- \| --- \| --- \| --- \| --- \| --- \| \| Number of comparisons per family \| 6 \|  \|  \|  \|  \|  \| \| Alpha \| 0.05 \|  \|  \|  \|  \|  \| \|  \|  \|  \|  \|  \|  \|  \| \| Tukey's multiple comparisons test \| Mean Diff. \| 95.00% CI of diff. \| Significant? \| Summary \| Adjusted P Value \|  \| \| Veh vs. Veh \| -69.40 \| -114.3 to -24.53 \| Yes \| *** \| 0.0006 \| A-B \| \| Veh vs. Ibr-1 \| -63.18 \| -108.1 to -18.31 \| Yes \| ** \| 0.0022 \| A-C \| \| Veh vs. Ibr-10 \| -18.80 \| -63.67 to 26.07 \| No \| ns \| 0.6928 \| A-D \| \| Veh vs. Ibr-1 \| 6.218 \| -38.65 to 51.09 \| No \| ns \| 0.9836 \| B-C \| \| Veh vs. Ibr-10 \| 50.60 \| 5.731 to 95.47 \| Yes \| * \| 0.0206 \| B-D \| \| Ibr-1 vs. Ibr-10 \| 44.38 \| -0.4870 to 89.26 \| No \| ns \| 0.0537 \| C-D \| |
| **Figure 1N. p-GSK3α/β fluorescence intensity in the Cortex** |
| \| Table Analyzed \| pGSK3a,b in cortex \| \| --- \| --- \| \| Data sets analyzed \| A-D \| \|  \|  \| \| ANOVA summary \|  \| \| F \| 29.82 \| \| P value \| <0.0001 \| \| P value summary \| **** \| \| Significant diff. among means (P < 0.05)? \| Yes \| \| R square \| 0.5013 \|  \| Number of families \| 1 \|  \|  \|  \|  \|  \| \| --- \| --- \| --- \| --- \| --- \| --- \| --- \| \| Number of comparisons per family \| 6 \|  \|  \|  \|  \|  \| \| Alpha \| 0.05 \|  \|  \|  \|  \|  \| \|  \|  \|  \|  \|  \|  \|  \| \| Tukey's multiple comparisons test \| Mean Diff. \| 95.00% CI of diff. \| Significant? \| Summary \| Adjusted P Value \|  \| \| Veh vs. Veh \| -229.8 \| -297.3 to -162.4 \| Yes \| **** \| <0.0001 \| A-B \| \| Veh vs. Ibr-1 \| -147.7 \| -215.1 to -80.27 \| Yes \| **** \| <0.0001 \| A-C \| \| Veh vs. Ibr-10 \| -64.52 \| -134.3 to 5.279 \| No \| ns \| 0.0806 \| A-D \| \| Veh vs. Ibr-1 \| 82.11 \| 14.67 to 149.5 \| Yes \| * \| 0.0105 \| B-C \| \| Veh vs. Ibr-10 \| 165.3 \| 95.49 to 235.1 \| Yes \| **** \| <0.0001 \| B-D \| \| Ibr-1 vs. Ibr-10 \| 83.18 \| 13.38 to 153.0 \| Yes \| * \| 0.0128 \| C-D \| |
| **Figure 1N. p-GSK3α/β fluorescence intensity in the hippocampal CA1** |
| \| Table Analyzed \| pGSK3a/b in CA1 \| \| --- \| --- \| \| Data sets analyzed \| A-D \| \|  \|  \| \| ANOVA summary \|  \| \| F \| 11.00 \| \| P value \| <0.0001 \| \| P value summary \| **** \| \| Significant diff. among means (P < 0.05)? \| Yes \| \| R square \| 0.2661 \|  \| Number of families \| 1 \|  \|  \|  \|  \|  \| \| --- \| --- \| --- \| --- \| --- \| --- \| --- \| \| Number of comparisons per family \| 6 \|  \|  \|  \|  \|  \| \| Alpha \| 0.05 \|  \|  \|  \|  \|  \| \|  \|  \|  \|  \|  \|  \|  \| \| Tukey's multiple comparisons test \| Mean Diff. \| 95.00% CI of diff. \| Significant? \| Summary \| Adjusted P Value \|  \| \| Veh vs. Veh \| -85.73 \| -133.4 to -38.07 \| Yes \| **** \| <0.0001 \| A-B \| \| Veh vs. Ibr-1 \| -57.77 \| -105.4 to -10.11 \| Yes \| * \| 0.0109 \| A-C \| \| Veh vs. Ibr-10 \| -1.851 \| -49.51 to 45.81 \| No \| ns \| 0.9996 \| A-D \| \| Veh vs. Ibr-1 \| 27.96 \| -19.19 to 75.11 \| No \| ns \| 0.4113 \| B-C \| \| Veh vs. Ibr-10 \| 83.88 \| 36.73 to 131.0 \| Yes \| **** \| <0.0001 \| B-D \| \| Ibr-1 vs. Ibr-10 \| 55.92 \| 8.773 to 103.1 \| Yes \| * \| 0.0133 \| C-D \| |
| **Figure 1P. Iba-1 fluorescence intensity in the Cortex** |
| \| Table Analyzed \| \| Iba-1 intensity \| \| \| --- \| --- \| --- \| --- \| \| Data sets analyzed \| \| A-D \| \| \|  \| \|  \| \| \| ANOVA summary \| \|  \| \| \| F \| \| 25.63 \| \| \| P value \| \| <0.0001 \| \| \| P value summary \| \| **** \| \| \| Significant diff. among means (P < 0.05)? \| \| Yes \| \| \| R square \| \| 0.4553 \| \| \| Number of families \| 1 \| \|  \| \|  \|  \|  \|  \| \| Number of comparisons per family \| 6 \| \|  \| \|  \|  \|  \|  \| \| Alpha \| 0.05 \| \|  \| \|  \|  \|  \|  \| \|  \|  \| \|  \| \|  \|  \|  \|  \| \| Tukey's multiple comparisons test \| Mean Diff. \| \| 95.00% CI of diff. \| \| Significant? \| Summary \| Adjusted P Value \|  \| \| Veh vs. Veh \| -150.1 \| \| -197.2 to -103.0 \| \| Yes \| **** \| <0.0001 \| A-B \| \| Veh vs. Ibr-1 \| -55.99 \| \| -103.1 to -8.865 \| \| Yes \| * \| 0.0131 \| A-C \| \| Veh vs. Ibr-10 \| -32.93 \| \| -80.05 to 14.20 \| \| No \| ns \| 0.2668 \| A-D \| \| Veh vs. Ibr-1 \| 94.13 \| \| 47.01 to 141.3 \| \| Yes \| **** \| <0.0001 \| B-C \| \| Veh vs. Ibr-10 \| 117.2 \| \| 70.07 to 164.3 \| \| Yes \| **** \| <0.0001 \| B-D \| \| Ibr-1 vs. Ibr-10 \| 23.06 \| \| -24.06 to 70.18 \| \| No \| ns \| 0.5776 \| C-D \| |
| **Figure 1P. Iba-1 fluorescence intensity in the hippocampal CA1** |
| \| Table Analyzed \| \| Iba-1 intensity \| \| \| --- \| --- \| --- \| --- \| \| Data sets analyzed \| \| F-I \| \| \|  \| \|  \| \| \| ANOVA summary \| \|  \| \| \| F \| \| 18.27 \| \| \| P value \| \| <0.0001 \| \| \| P value summary \| \| **** \| \| \| Significant diff. among means (P < 0.05)? \| \| Yes \| \| \| R square \| \| 0.3760 \| \| \| Number of families \| 1 \| \|  \| \|  \|  \|  \|  \| \| Number of comparisons per family \| 6 \| \|  \| \|  \|  \|  \|  \| \| Alpha \| 0.05 \| \|  \| \|  \|  \|  \|  \| \|  \|  \| \|  \| \|  \|  \|  \|  \| \| Tukey's multiple comparisons test \| Mean Diff. \| \| 95.00% CI of diff. \| \| Significant? \| Summary \| Adjusted P Value \|  \| \| Veh vs. Veh \| -146.4 \| \| -202.2 to -90.53 \| \| Yes \| **** \| <0.0001 \| F-G \| \| Veh vs. Ibr-1 \| -75.30 \| \| -131.2 to -19.44 \| \| Yes \| ** \| 0.0036 \| F-H \| \| Veh vs. Ibr-10 \| -24.54 \| \| -81.00 to 31.92 \| \| No \| ns \| 0.6675 \| F-I \| \| Veh vs. Ibr-1 \| 71.09 \| \| 15.23 to 126.9 \| \| Yes \| ** \| 0.0068 \| G-H \| \| Veh vs. Ibr-10 \| 121.9 \| \| 65.39 to 178.3 \| \| Yes \| **** \| <0.0001 \| G-I \| \| Ibr-1 vs. Ibr-10 \| 50.76 \| \| -5.701 to 107.2 \| \| No \| ns \| 0.0938 \| H-I \| |
| **Figure 1P. Iba-1 fluorescence intensity in the hippocampal DG** |
| \| Table Analyzed \| \| Iba-1 intensity \| \| \| --- \| --- \| --- \| --- \| \| Data sets analyzed \| \| K-N \| \| \|  \| \|  \| \| \| ANOVA summary \| \|  \| \| \| F \| \| 18.88 \| \| \| P value \| \| <0.0001 \| \| \| P value summary \| \| **** \| \| \| Significant diff. among means (P < 0.05)? \| \| Yes \| \| \| R square \| \| 0.3836 \| \| \| Number of families \| 1 \| \|  \| \|  \|  \|  \|  \| \| Number of comparisons per family \| 6 \| \|  \| \|  \|  \|  \|  \| \| Alpha \| 0.05 \| \|  \| \|  \|  \|  \|  \| \|  \|  \| \|  \| \|  \|  \|  \|  \| \| Tukey's multiple comparisons test \| Mean Diff. \| \| 95.00% CI of diff. \| \| Significant? \| Summary \| Adjusted P Value \|  \| \| Veh vs. Veh \| -116.7 \| \| -164.3 to -69.06 \| \| Yes \| **** \| <0.0001 \| K-L \| \| Veh vs. Ibr-1 \| -92.56 \| \| -140.2 to -44.97 \| \| Yes \| **** \| <0.0001 \| K-M \| \| Veh vs. Ibr-10 \| -20.22 \| \| -68.34 to 27.89 \| \| No \| ns \| 0.6904 \| K-N \| \| Veh vs. Ibr-1 \| 24.10 \| \| -23.50 to 71.70 \| \| No \| ns \| 0.5495 \| L-M \| \| Veh vs. Ibr-10 \| 96.44 \| \| 48.33 to 144.6 \| \| Yes \| **** \| <0.0001 \| L-N \| \| Ibr-1 vs. Ibr-10 \| 72.34 \| \| 24.23 to 120.5 \| \| Yes \| *** \| 0.0009 \| M-N \| |
| **Figure 1P. Iba-1 labeled area in the Cortex** |
| \| Table Analyzed \| \| Iba-1 area \| \| \| --- \| --- \| --- \| --- \| \| Data sets analyzed \| \| A-D \| \| \|  \| \|  \| \| \| ANOVA summary \| \|  \| \| \| F \| \| 34.76 \| \| \| P value \| \| <0.0001 \| \| \| P value summary \| \| **** \| \| \| Significant diff. among means (P < 0.05)? \| \| Yes \| \| \| R square \| \| 0.5313 \| \| \| Number of families \| 1 \| \|  \| \|  \|  \|  \|  \| \| Number of comparisons per family \| 6 \| \|  \| \|  \|  \|  \|  \| \| Alpha \| 0.05 \| \|  \| \|  \|  \|  \|  \| \|  \|  \| \|  \| \|  \|  \|  \|  \| \| Tukey's multiple comparisons test \| Mean Diff. \| \| 95.00% CI of diff. \| \| Significant? \| Summary \| Adjusted P Value \|  \| \| Veh vs. Veh \| -2.371 \| \| -2.986 to -1.756 \| \| Yes \| **** \| <0.0001 \| A-B \| \| Veh vs. Ibr-1 \| -1.162 \| \| -1.778 to -0.5471 \| \| Yes \| **** \| <0.0001 \| A-C \| \| Veh vs. Ibr-10 \| -0.8687 \| \| -1.484 to -0.2535 \| \| Yes \| ** \| 0.0021 \| A-D \| \| Veh vs. Ibr-1 \| 1.208 \| \| 0.5933 to 1.824 \| \| Yes \| **** \| <0.0001 \| B-C \| \| Veh vs. Ibr-10 \| 1.502 \| \| 0.8869 to 2.117 \| \| Yes \| **** \| <0.0001 \| B-D \| \| Ibr-1 vs. Ibr-10 \| 0.2937 \| \| -0.3215 to 0.9089 \| \| No \| ns \| 0.5975 \| C-D \| |
| **Figure 1P. Iba-1 labeled area in the hippocampal CA1** |
| \| Table Analyzed \| \| Iba-1 area \| \| \| --- \| --- \| --- \| --- \| \| Data sets analyzed \| \| F-I \| \| \|  \| \|  \| \| \| ANOVA summary \| \|  \| \| \| F \| \| 19.24 \| \| \| P value \| \| <0.0001 \| \| \| P value summary \| \| **** \| \| \| Significant diff. among means (P < 0.05)? \| \| Yes \| \| \| R square \| \| 0.3881 \| \| \| Number of families \| 1 \| \|  \| \|  \|  \|  \|  \| \| Number of comparisons per family \| 6 \| \|  \| \|  \|  \|  \|  \| \| Alpha \| 0.05 \| \|  \| \|  \|  \|  \|  \| \|  \|  \| \|  \| \|  \|  \|  \|  \| \| Tukey's multiple comparisons test \| Mean Diff. \| \| 95.00% CI of diff. \| \| Significant? \| Summary \| Adjusted P Value \|  \| \| Veh vs. Veh \| -3.029 \| \| -4.117 to -1.941 \| \| Yes \| **** \| <0.0001 \| F-G \| \| Veh vs. Ibr-1 \| -1.687 \| \| -2.775 to -0.5983 \| \| Yes \| *** \| 0.0006 \| F-H \| \| Veh vs. Ibr-10 \| -0.8450 \| \| -1.945 to 0.2551 \| \| No \| ns \| 0.1918 \| F-I \| \| Veh vs. Ibr-1 \| 1.342 \| \| 0.2541 to 2.431 \| \| Yes \| ** \| 0.0092 \| G-H \| \| Veh vs. Ibr-10 \| 2.184 \| \| 1.084 to 3.284 \| \| Yes \| **** \| <0.0001 \| G-I \| \| Ibr-1 vs. Ibr-10 \| 0.8416 \| \| -0.2585 to 1.942 \| \| No \| ns \| 0.1948 \| H-I \| |
| **Figure 1P. Iba-1 labeled area in the hippocampal DG** |
| \| Table Analyzed \| \| Iba-1 area \| \| \| --- \| --- \| --- \| --- \| \| Data sets analyzed \| \| K-N \| \| \|  \| \|  \| \| \| ANOVA summary \| \|  \| \| \| F \| \| 21.47 \| \| \| P value \| \| <0.0001 \| \| \| P value summary \| \| **** \| \| \| Significant diff. among means (P < 0.05)? \| \| Yes \| \| \| R square \| \| 0.4144 \| \| \| Number of families \| 1 \| \|  \| \|  \|  \|  \|  \| \| Number of comparisons per family \| 6 \| \|  \| \|  \|  \|  \|  \| \| Alpha \| 0.05 \| \|  \| \|  \|  \|  \|  \| \|  \|  \| \|  \| \|  \|  \|  \|  \| \| Tukey's multiple comparisons test \| Mean Diff. \| \| 95.00% CI of diff. \| \| Significant? \| Summary \| Adjusted P Value \|  \| \| Veh vs. Veh \| -4.179 \| \| -5.747 to -2.611 \| \| Yes \| **** \| <0.0001 \| K-L \| \| Veh vs. Ibr-1 \| -3.741 \| \| -5.309 to -2.173 \| \| Yes \| **** \| <0.0001 \| K-M \| \| Veh vs. Ibr-10 \| -1.462 \| \| -3.047 to 0.1229 \| \| No \| ns \| 0.0816 \| K-N \| \| Veh vs. Ibr-1 \| 0.4384 \| \| -1.130 to 2.006 \| \| No \| ns \| 0.8841 \| L-M \| \| Veh vs. Ibr-10 \| 2.717 \| \| 1.132 to 4.302 \| \| Yes \| *** \| 0.0001 \| L-N \| \| Ibr-1 vs. Ibr-10 \| 2.279 \| \| 0.6940 to 3.864 \| \| Yes \| ** \| 0.0017 \| M-N \| |
| **Figure 1R. GFAP fluorescence intensity in the Cortex** |
| \| Table Analyzed \| \| GFAP intensity \| \| \| --- \| --- \| --- \| --- \| \| Data sets analyzed \| \| A-D \| \| \|  \| \|  \| \| \| ANOVA summary \| \|  \| \| \| F \| \| 70.75 \| \| \| P value \| \| <0.0001 \| \| \| P value summary \| \| **** \| \| \| Significant diff. among means (P < 0.05)? \| \| Yes \| \| \| R square \| \| 0.6976 \| \| \| Number of families \| 1 \| \|  \| \|  \|  \|  \|  \| \| Number of comparisons per family \| 6 \| \|  \| \|  \|  \|  \|  \| \| Alpha \| 0.05 \| \|  \| \|  \|  \|  \|  \| \|  \|  \| \|  \| \|  \|  \|  \|  \| \| Tukey's multiple comparisons test \| Mean Diff. \| \| 95.00% CI of diff. \| \| Significant? \| Summary \| Adjusted P Value \|  \| \| Veh vs. Veh \| -4202 \| \| -5003 to -3400 \| \| Yes \| **** \| <0.0001 \| A-B \| \| Veh vs. Ibr-1 \| -3388 \| \| -4190 to -2586 \| \| Yes \| **** \| <0.0001 \| A-C \| \| Veh vs. Ibr-10 \| -2333 \| \| -3134 to -1531 \| \| Yes \| **** \| <0.0001 \| A-D \| \| Veh vs. Ibr-1 \| 813.7 \| \| 12.02 to 1615 \| \| Yes \| * \| 0.0453 \| B-C \| \| Veh vs. Ibr-10 \| 1869 \| \| 1068 to 2671 \| \| Yes \| **** \| <0.0001 \| B-D \| \| Ibr-1 vs. Ibr-10 \| 1055 \| \| 253.8 to 1857 \| \| Yes \| ** \| 0.0047 \| C-D \| |
| **Figure 1R. GFAP fluorescence intensity in the hippocampal CA1** |
| \| Table Analyzed \| \| GFAP intensity \| \| \| --- \| --- \| --- \| --- \| \| Data sets analyzed \| \| F-I \| \| \|  \| \|  \| \| \| ANOVA summary \| \|  \| \| \| F \| \| 42.07 \| \| \| P value \| \| <0.0001 \| \| \| P value summary \| \| **** \| \| \| Significant diff. among means (P < 0.05)? \| \| Yes \| \| \| R squared \| \| 0.5784 \| \| \| Number of families \| 1 \| \|  \| \|  \|  \|  \|  \| \| Number of comparisons per family \| 6 \| \|  \| \|  \|  \|  \|  \| \| Alpha \| 0.05 \| \|  \| \|  \|  \|  \|  \| \|  \|  \| \|  \| \|  \|  \|  \|  \| \| Tukey's multiple comparisons test \| Mean Diff. \| \| 95.00% CI of diff. \| \| Below threshold? \| Summary \| Adjusted P Value \|  \| \| - vs. - \| -245.0 \| \| -304.6 to -185.4 \| \| Yes \| **** \| <0.0001 \| F-G \| \| - vs. 1 \| -174.6 \| \| -234.2 to -115.0 \| \| Yes \| **** \| <0.0001 \| F-H \| \| - vs. 10 \| -105.0 \| \| -164.6 to -45.42 \| \| Yes \| **** \| <0.0001 \| F-I \| \| - vs. 1 \| 70.41 \| \| 10.81 to 130.0 \| \| Yes \| * \| 0.0138 \| G-H \| \| - vs. 10 \| 140.0 \| \| 80.40 to 199.6 \| \| Yes \| **** \| <0.0001 \| G-I \| \| 1 vs. 10 \| 69.59 \| \| 9.994 to 129.2 \| \| Yes \| * \| 0.0153 \| H-I \| |
| **Figure 1R. GFAP fluorescence intensity in the hippocampal DG** |
| \| Table Analyzed \| \| GFAP intensity \| \| \| --- \| --- \| --- \| --- \| \| Data sets analyzed \| \| K-N \| \| \|  \| \|  \| \| \| ANOVA summary \| \|  \| \| \| F \| \| 54.67 \| \| \| P value \| \| <0.0001 \| \| \| P value summary \| \| **** \| \| \| Significant diff. among means (P < 0.05)? \| \| Yes \| \| \| R squared \| \| 0.6406 \| \| \| Number of families \| 1 \| \|  \| \|  \|  \|  \|  \| \| Number of comparisons per family \| 6 \| \|  \| \|  \|  \|  \|  \| \| Alpha \| 0.05 \| \|  \| \|  \|  \|  \|  \| \|  \|  \| \|  \| \|  \|  \|  \|  \| \| Tukey's multiple comparisons test \| Mean Diff. \| \| 95.00% CI of diff. \| \| Below threshold? \| Summary \| Adjusted P Value \|  \| \| - vs. - \| -333.0 \| \| -410.1 to -255.9 \| \| Yes \| **** \| <0.0001 \| K-L \| \| - vs. 1 \| -317.9 \| \| -395.0 to -240.8 \| \| Yes \| **** \| <0.0001 \| K-M \| \| - vs. 10 \| -190.6 \| \| -267.7 to -113.5 \| \| Yes \| **** \| <0.0001 \| K-N \| \| - vs. 1 \| 15.12 \| \| -62.00 to 92.25 \| \| No \| ns \| 0.9558 \| L-M \| \| - vs. 10 \| 142.4 \| \| 65.29 to 219.5 \| \| Yes \| **** \| <0.0001 \| L-N \| \| 1 vs. 10 \| 127.3 \| \| 50.16 to 204.4 \| \| Yes \| *** \| 0.0002 \| M-N \| |
| **Figure 1R. GFAP labeled area in the Cortex** |
| \| Table Analyzed \| \| GFAP area \| \| \| --- \| --- \| --- \| --- \| \| Data sets analyzed \| \| A-D \| \| \|  \| \|  \| \| \| ANOVA summary \| \|  \| \| \| F \| \| 59.89 \| \| \| P value \| \| <0.0001 \| \| \| P value summary \| \| **** \| \| \| Significant diff. among means (P < 0.05)? \| \| Yes \| \| \| R square \| \| 0.6614 \| \| \| Number of families \| 1 \| \|  \| \|  \|  \|  \|  \| \| Number of comparisons per family \| 6 \| \|  \| \|  \|  \|  \|  \| \| Alpha \| 0.05 \| \|  \| \|  \|  \|  \|  \| \|  \|  \| \|  \| \|  \|  \|  \|  \| \| Tukey's multiple comparisons test \| Mean Diff. \| \| 95.00% CI of diff. \| \| Significant? \| Summary \| Adjusted P Value \|  \| \| Veh vs. Veh \| -10.15 \| \| -12.25 to -8.061 \| \| Yes \| **** \| <0.0001 \| A-B \| \| Veh vs. Ibr-1 \| -8.011 \| \| -10.10 to -5.918 \| \| Yes \| **** \| <0.0001 \| A-C \| \| Veh vs. Ibr-10 \| -5.563 \| \| -7.656 to -3.470 \| \| Yes \| **** \| <0.0001 \| A-D \| \| Veh vs. Ibr-1 \| 2.144 \| \| 0.05071 to 4.236 \| \| Yes \| * \| 0.0426 \| B-C \| \| Veh vs. Ibr-10 \| 4.591 \| \| 2.498 to 6.684 \| \| Yes \| **** \| <0.0001 \| B-D \| \| Ibr-1 vs. Ibr-10 \| 2.447 \| \| 0.3544 to 4.540 \| \| Yes \| * \| 0.0151 \| C-D \| |
| **Figure 1R. GFAP labeled area in the hippocampal CA1** |
| \| Table Analyzed \| \| GFAP area \| \| \| --- \| --- \| --- \| --- \| \| Data sets analyzed \| \| F-I \| \| \|  \| \|  \| \| \| ANOVA summary \| \|  \| \| \| F \| \| 38.44 \| \| \| P value \| \| <0.0001 \| \| \| P value summary \| \| **** \| \| \| Significant diff. among means (P < 0.05)? \| \| Yes \| \| \| R squared \| \| 0.5562 \| \| \| Number of families \| 1 \| \|  \| \|  \|  \|  \|  \| \| Number of comparisons per family \| 6 \| \|  \| \|  \|  \|  \|  \| \| Alpha \| 0.05 \| \|  \| \|  \|  \|  \|  \| \|  \|  \| \|  \| \|  \|  \|  \|  \| \| Tukey's multiple comparisons test \| Mean Diff. \| \| 95.00% CI of diff. \| \| Below threshold? \| Summary \| Adjusted P Value \|  \| \| - vs. - \| -8.890 \| \| -11.20 to -6.583 \| \| Yes \| **** \| <0.0001 \| F-G \| \| - vs. 1 \| -6.298 \| \| -8.605 to -3.992 \| \| Yes \| **** \| <0.0001 \| F-H \| \| - vs. 10 \| -3.064 \| \| -5.370 to -0.7571 \| \| Yes \| ** \| 0.0043 \| F-I \| \| - vs. 1 \| 2.592 \| \| 0.2850 to 4.898 \| \| Yes \| * \| 0.0212 \| G-H \| \| - vs. 10 \| 5.826 \| \| 3.519 to 8.133 \| \| Yes \| **** \| <0.0001 \| G-I \| \| 1 vs. 10 \| 3.234 \| \| 0.9279 to 5.541 \| \| Yes \| ** \| 0.0023 \| H-I \| |
| **Figure 1R. GFAP labeled area in the hippocampal DG** |
| \| Table Analyzed \| \| GFAP area \| \| \| --- \| --- \| --- \| --- \| \| Data sets analyzed \| \| K-N \| \| \|  \| \|  \| \| \| ANOVA summary \| \|  \| \| \| F \| \| 46.82 \| \| \| P value \| \| <0.0001 \| \| \| P value summary \| \| **** \| \| \| Significant diff. among means (P < 0.05)? \| \| Yes \| \| \| R squared \| \| 0.6042 \| \| \| Number of families \| 1 \| \|  \| \|  \|  \|  \|  \| \| Number of comparisons per family \| 6 \| \|  \| \|  \|  \|  \|  \| \| Alpha \| 0.05 \| \|  \| \|  \|  \|  \|  \| \|  \|  \| \|  \| \|  \|  \|  \|  \| \| Tukey's multiple comparisons test \| Mean Diff. \| \| 95.00% CI of diff. \| \| Below threshold? \| Summary \| Adjusted P Value \|  \| \| - vs. - \| -12.10 \| \| -15.16 to -9.034 \| \| Yes \| **** \| <0.0001 \| K-L \| \| - vs. 1 \| -11.74 \| \| -14.80 to -8.673 \| \| Yes \| **** \| <0.0001 \| K-M \| \| - vs. 10 \| -6.529 \| \| -9.592 to -3.466 \| \| Yes \| **** \| <0.0001 \| K-N \| \| - vs. 1 \| 0.3610 \| \| -2.702 to 3.424 \| \| No \| Ns \| 0.9898 \| L-M \| \| - vs. 10 \| 5.567 \| \| 2.505 to 8.630 \| \| Yes \| **** \| <0.0001 \| L-N \| \| 1 vs. 10 \| 5.206 \| \| 2.144 to 8.269 \| \| Yes \| *** \| 0.0001 \| M-N \| |
| **Figure 1T. NLRP3 fluorescence intensity in the Cortex** |
| \| Table Analyzed \| NLRP3- cortex \| \| --- \| --- \| \| Data sets analyzed \| A-D \| \|  \|  \| \| ANOVA summary \|  \| \| F \| 26.33 \| \| P value \| <0.0001 \| \| P value summary \| **** \| \| Significant diff. among means (P < 0.05)? \| Yes \| \| R square \| 0.4646 \|  \| Number of families \| 1 \|  \|  \|  \|  \|  \| \| --- \| --- \| --- \| --- \| --- \| --- \| --- \| \| Number of comparisons per family \| 6 \|  \|  \|  \|  \|  \| \| Alpha \| 0.05 \|  \|  \|  \|  \|  \| \|  \|  \|  \|  \|  \|  \|  \| \| Tukey's multiple comparisons test \| Mean Diff. \| 95.00% CI of diff. \| Significant? \| Summary \| Adjusted P Value \|  \| \| Veh vs. Veh \| -137.2 \| -184.1 to -90.32 \| Yes \| **** \| <0.0001 \| A-B \| \| Veh vs. Ibr-1 \| -25.56 \| -72.47 to 21.35 \| No \| ns \| 0.4866 \| A-C \| \| Veh vs. Ibr-10 \| -4.743 \| -51.65 to 42.17 \| No \| ns \| 0.9935 \| A-D \| \| Veh vs. Ibr-1 \| 111.7 \| 65.26 to 158.1 \| Yes \| **** \| <0.0001 \| B-C \| \| Veh vs. Ibr-10 \| 132.5 \| 86.08 to 178.9 \| Yes \| **** \| <0.0001 \| B-D \| \| Ibr-1 vs. Ibr-10 \| 20.82 \| -25.59 to 67.22 \| No \| ns \| 0.6448 \| C-D \| |
| **Figure 1T. NLRP3 fluorescence intensity in the hippocampal CA1** |
| \| Table Analyzed \| NLRP3-CA1 \| \| --- \| --- \| \| Data sets analyzed \| A-D \| \|  \|  \| \| ANOVA summary \|  \| \| F \| 7.061 \| \| P value \| 0.0003 \| \| P value summary \| *** \| \| Significant diff. among means (P < 0.05)? \| Yes \| \| R square \| 0.1888 \|  \| Number of families \| 1 \|  \|  \|  \|  \|  \| \| --- \| --- \| --- \| --- \| --- \| --- \| --- \| \| Number of comparisons per family \| 6 \|  \|  \|  \|  \|  \| \| Alpha \| 0.05 \|  \|  \|  \|  \|  \| \|  \|  \|  \|  \|  \|  \|  \| \| Tukey's multiple comparisons test \| Mean Diff. \| 95.00% CI of diff. \| Significant? \| Summary \| Adjusted P Value \|  \| \| Veh vs. Veh \| -46.81 \| -77.24 to -16.38 \| Yes \| *** \| 0.0007 \| A-B \| \| Veh vs. Ibr-1 \| -3.739 \| -34.17 to 26.69 \| No \| ns \| 0.9884 \| A-C \| \| Veh vs. Ibr-10 \| -8.146 \| -38.57 to 22.28 \| No \| ns \| 0.8966 \| A-D \| \| Veh vs. Ibr-1 \| 43.07 \| 12.97 to 73.17 \| Yes \| ** \| 0.0018 \| B-C \| \| Veh vs. Ibr-10 \| 38.67 \| 8.563 to 68.77 \| Yes \| ** \| 0.0061 \| B-D \| \| Ibr-1 vs. Ibr-10 \| -4.407 \| -34.51 to 25.69 \| No \| ns \| 0.9807 \| C-D \| \|  \|  \|  \|  \|  \|  \|  \| |
| **Figure 1U. NLRP3 mRNA levels in the Cortex** |
| \| Table Analyzed \| NLRP3 \| \| --- \| --- \| \|  \|  \| \| Brown-Forsythe ANOVA test \|  \| \| F* (DFn, DFd) \| 8.178 (3.000, 8.153) \| \| P value \| 0.0077 \| \| P value summary \| ** \| \| Significant diff. among means (P < 0.05)? \| Yes \|  \| Number of families \| 1 \|  \|  \|  \|  \|  \| \| --- \| --- \| --- \| --- \| --- \| --- \| --- \| \| Number of comparisons per family \| 6 \|  \|  \|  \|  \|  \| \| Alpha \| 0.05 \|  \|  \|  \|  \|  \| \|  \|  \|  \|  \|  \|  \|  \| \| Dunnett's T3 multiple comparisons test \| Mean Diff. \| 95.00% CI of diff. \| Below threshold? \| Summary \| Adjusted P Value \|  \| \| - vs. - \| -1.289 \| -1.838 to -0.7399 \| Yes \| *** \| 0.0002 \| A-B \| \| - vs. 1 \| -0.7726 \| -2.099 to 0.5533 \| No \| ns \| 0.2784 \| A-C \| \| - vs. 10 \| -0.5978 \| -1.195 to -0.0003139 \| Yes \| * \| 0.0499 \| A-D \| \| - vs. 1 \| 0.5164 \| -0.8237 to 1.856 \| No \| ns \| 0.6108 \| B-C \| \| - vs. 10 \| 0.6912 \| 0.07096 to 1.311 \| Yes \| * \| 0.0291 \| B-D \| \| 1 vs. 10 \| 0.1748 \| -1.185 to 1.534 \| No \| ns \| 0.9935 \| C-D \| |
| **Figure 1U. NLRP3 mRNA levels in the Hippocampus** |
| \| Table Analyzed \| NLRP3 \| \| --- \| --- \| \| Data sets analyzed \| F-I \| \|  \|  \| \| ANOVA summary \|  \| \| F \| 19.61 \| \| P value \| <0.0001 \| \| P value summary \| **** \| \| Significant diff. among means (P < 0.05)? \| Yes \| \| R squared \| 0.7463 \|  \| Number of families \| 1 \|  \|  \|  \|  \|  \| \| --- \| --- \| --- \| --- \| --- \| --- \| --- \| \| Number of comparisons per family \| 6 \|  \|  \|  \|  \|  \| \| Alpha \| 0.05 \|  \|  \|  \|  \|  \| \|  \|  \|  \|  \|  \|  \|  \| \| Tukey's multiple comparisons test \| Mean Diff. \| 95.00% CI of diff. \| Below threshold? \| Summary \| Adjusted P Value \|  \| \| - vs. - \| -2.402 \| -3.477 to -1.327 \| Yes \| **** \| <0.0001 \| F-G \| \| - vs. 1 \| -2.272 \| -3.347 to -1.197 \| Yes \| **** \| <0.0001 \| F-H \| \| - vs. 10 \| -0.5996 \| -1.675 to 0.4754 \| No \| ns \| 0.4220 \| F-I \| \| - vs. 1 \| 0.1295 \| -0.9455 to 1.204 \| No \| ns \| 0.9864 \| G-H \| \| - vs. 10 \| 1.802 \| 0.7273 to 2.877 \| Yes \| *** \| 0.0007 \| G-I \| \| 1 vs. 10 \| 1.673 \| 0.5978 to 2.748 \| Yes \| ** \| 0.0016 \| H-I \| |
| **Supplementary Figure 1A. Spontaneous alternations in Y maze** |
| \| Table Analyzed \| Y maze \| \| --- \| --- \| \| Data sets analyzed \| A-C \| \|  \|  \| \| ANOVA summary \|  \| \| F \| 1.866 \| \| P value \| 0.1807 \| \| P value summary \| ns \| \| Significant diff. among means (P < 0.05)? \| No \| \| R squared \| 0.1572 \|  \| Number of families \| 1 \|  \|  \|  \|  \|  \| \| --- \| --- \| --- \| --- \| --- \| --- \| --- \| \| Number of comparisons per family \| 3 \|  \|  \|  \|  \|  \| \| Alpha \| 0.05 \|  \|  \|  \|  \|  \| \|  \|  \|  \|  \|  \|  \|  \| \| Tukey's multiple comparisons test \| Mean Diff. \| 95.00% CI of diff. \| Below threshold? \| Summary \| Adjusted P Value \|  \| \| Column A vs. Column B \| 1.448 \| -12.80 to 15.70 \| No \| ns \| 0.9643 \| A-B \| \| Column A vs. Column C \| 9.895 \| -4.356 to 24.15 \| No \| ns \| 0.2096 \| A-C \| \| Column B vs. Column C \| 8.446 \| -5.321 to 22.21 \| No \| ns \| 0.2889 \| B-C \| |
| **Supplementary Figure 1A. Total arm entry in Y maze** |
| \| Table Analyzed \| Y maze \| \| --- \| --- \| \| Data sets analyzed \| A-C \| \|  \|  \| \| ANOVA summary \|  \| \| F \| 0.8713 \| \| P value \| 0.4337 \| \| P value summary \| ns \| \| Significant diff. among means (P < 0.05)? \| No \| \| R squared \| 0.08015 \|  \| Number of families \| 1 \|  \|  \|  \|  \|  \| \| --- \| --- \| --- \| --- \| --- \| --- \| --- \| \| Number of comparisons per family \| 3 \|  \|  \|  \|  \|  \| \| Alpha \| 0.05 \|  \|  \|  \|  \|  \| \|  \|  \|  \|  \|  \|  \|  \| \| Tukey's multiple comparisons test \| Mean Diff. \| 95.00% CI of diff. \| Below threshold? \| Summary \| Adjusted P Value \|  \| \| Column A vs. Column B \| -2.286 \| -10.03 to 5.455 \| No \| ns \| 0.7388 \| A-B \| \| Column A vs. Column C \| -4.036 \| -11.78 to 3.705 \| No \| ns \| 0.4014 \| A-C \| \| Column B vs. Column C \| -1.750 \| -9.228 to 5.728 \| No \| ns \| 0.8259 \| B-C \| |
| **Supplementary Figure 1B. Object preference in NOR training** |
| \| Table Analyzed \| NOR training \| \| --- \| --- \| \| Data sets analyzed \| A-C \| \|  \|  \| \| ANOVA summary \|  \| \| F \| 0.6909 \| \| P value \| 0.5127 \| \| P value summary \| ns \| \| Significant diff. among means (P < 0.05)? \| No \| \| R squared \| 0.06462 \|  \| Number of families \| 1 \|  \|  \|  \|  \|  \| \| --- \| --- \| --- \| --- \| --- \| --- \| --- \| \| Number of comparisons per family \| 3 \|  \|  \|  \|  \|  \| \| Alpha \| 0.05 \|  \|  \|  \|  \|  \| \|  \|  \|  \|  \|  \|  \|  \| \| Tukey's multiple comparisons test \| Mean Diff. \| 95.00% CI of diff. \| Below threshold? \| Summary \| Adjusted P Value \|  \| \| Column A vs. Column B \| 8.953 \| -14.04 to 31.94 \| No \| ns \| 0.5943 \| A-B \| \| Column A vs. Column C \| -0.1528 \| -23.14 to 22.84 \| No \| ns \| 0.9998 \| A-C \| \| Column B vs. Column C \| -9.106 \| -31.32 to 13.10 \| No \| ns \| 0.5627 \| B-C \| |
| **Supplementary Figure 1B. Novelty preference in NOR test** |
| \| Table Analyzed \| NOR test \| \| --- \| --- \| \| Data sets analyzed \| A-C \| \|  \|  \| \| ANOVA summary \|  \| \| F \| 0.07086 \| \| P value \| 0.9318 \| \| P value summary \| ns \| \| Significant diff. among means (P < 0.05)? \| No \| \| R squared \| 0.007036 \|  \| Number of families \| 1 \|  \|  \|  \|  \|  \| \| --- \| --- \| --- \| --- \| --- \| --- \| --- \| \| Number of comparisons per family \| 3 \|  \|  \|  \|  \|  \| \| Alpha \| 0.05 \|  \|  \|  \|  \|  \| \|  \|  \|  \|  \|  \|  \|  \| \| Tukey's multiple comparisons test \| Mean Diff. \| 95.00% CI of diff. \| Below threshold? \| Summary \| Adjusted P Value \|  \| \| Column A vs. Column B \| 1.991 \| -18.42 to 22.40 \| No \| ns \| 0.9670 \| A-B \| \| Column A vs. Column C \| -0.8771 \| -21.28 to 19.53 \| No \| ns \| 0.9935 \| A-C \| \| Column B vs. Column C \| -2.868 \| -22.58 to 16.85 \| No \| ns \| 0.9283 \| B-C \| |
| **Supplementary Figure 2A. ALT activity in Plasma** |
| \| Table Analyzed \| ALTactivity \| \| --- \| --- \| \| Data sets analyzed \| A-C \| \|  \|  \| \| ANOVA summary \|  \| \| F \| 0.9445 \| \| P value \| 0.4055 \| \| P value summary \| ns \| \| Significant diff. among means (P < 0.05)? \| No \| \| R squared \| 0.08630 \|  \| Number of families \| 1 \|  \|  \|  \|  \|  \| \| --- \| --- \| --- \| --- \| --- \| --- \| --- \| \| Number of comparisons per family \| 3 \|  \|  \|  \|  \|  \| \| Alpha \| 0.05 \|  \|  \|  \|  \|  \| \|  \|  \|  \|  \|  \|  \|  \| \| Tukey's multiple comparisons test \| Mean Diff. \| 95.00% CI of diff. \| Below threshold? \| Summary \| Adjusted P Value \|  \| \| Column A vs. Column B \| 1.976 \| -6.494 to 10.45 \| No \| ns \| 0.8269 \| A-B \| \| Column A vs. Column C \| -2.461 \| -10.93 to 6.008 \| No \| ns \| 0.7458 \| A-C \| \| Column B vs. Column C \| -4.437 \| -12.62 to 3.745 \| No \| ns \| 0.3739 \| B-C \| |
| **Supplementary Figure 2B. Cleaved caspase-3 levels in the Liver** |
| \| \| \| Table Analyzed \| C.caspase-3 \| \| --- \| --- \| \| Data sets analyzed \| A-C \| \|  \|  \| \| ANOVA summary \|  \| \| F \| 1.720 \| \| P value \| 0.2073 \| \| P value summary \| ns \| \| Significant diff. among means (P < 0.05)? \| No \| \| R squared \| 0.1604 \| \|  \| \| --- \| --- \| --- \| --- \| --- \| --- \| --- \| --- \| --- \| --- \| --- \| --- \| --- \| --- \| --- \| --- \| --- \| --- \| --- \| --- \| \| \| Number of families \| 1 \|  \|  \|  \|  \|  \| \| --- \| --- \| --- \| --- \| --- \| --- \| --- \| \| Number of comparisons per family \| 3 \|  \|  \|  \|  \|  \| \| Alpha \| 0.05 \|  \|  \|  \|  \|  \| \|  \|  \|  \|  \|  \|  \|  \| \| Tukey's multiple comparisons test \| Mean Diff. \| 95.00% CI of diff. \| Below threshold? \| Summary \| Adjusted P Value \|  \| \| Column A vs. Column B \| -34.62 \| -92.09 to 22.85 \| No \| ns \| 0.2977 \| A-B \| \| Column A vs. Column C \| 2.919 \| -54.55 to 60.39 \| No \| ns \| 0.9908 \| A-C \| \| Column B vs. Column C \| 37.54 \| -19.93 to 95.01 \| No \| ns \| 0.2447 \| B-C \| \|  \| \|  \|  \| \|  \|  \|  \|  \|  \|  \| \| --- \| --- \| --- \| --- \| --- \| --- \| --- \| --- \| --- \| --- \| --- \| --- \| --- \| --- \| --- \| --- \| --- \| --- \| --- \| --- \| --- \| --- \| --- \| --- \| --- \| --- \| --- \| --- \| --- \| --- \| --- \| --- \| --- \| --- \| --- \| --- \| --- \| --- \| --- \| --- \| --- \| --- \| --- \| --- \| --- \| --- \| --- \| --- \| --- \| --- \| --- \| --- \| --- \| --- \| --- \| --- \| --- \| --- \| --- \| --- \| --- \| --- \| --- \| --- \| --- \| --- \| --- \| --- \| --- \| --- \| --- \| --- \| --- \| --- \| --- \| --- \| --- \| --- \| --- \| --- \| --- \| --- \| --- \| --- \| --- \| --- \| --- \| |
| **Supplementary Figure 3A. Number of Iba-1 positive cell in the Cortex** |
| \| Table Analyzed \| \| Iba-1 cell number \| \| \| --- \| --- \| --- \| --- \| \| Data sets analyzed \| \| A-D \| \| \|  \| \|  \| \| \| ANOVA summary \| \|  \| \| \| F \| \| 22.26 \| \| \| P value \| \| <0.0001 \| \| \| P value summary \| \| **** \| \| \| Significant diff. among means (P < 0.05)? \| \| Yes \| \| \| R square \| \| 0.4205 \| \| \| Number of families \| 1 \| \|  \| \|  \|  \|  \|  \| \| Number of comparisons per family \| 6 \| \|  \| \|  \|  \|  \|  \| \| Alpha \| 0.05 \| \|  \| \|  \|  \|  \|  \| \|  \|  \| \|  \| \|  \|  \|  \|  \| \| Tukey's multiple comparisons test \| Mean Diff. \| \| 95.00% CI of diff. \| \| Significant? \| Summary \| Adjusted P Value \|  \| \| Veh vs. Veh \| -239.2 \| \| -316.0 to -162.4 \| \| Yes \| **** \| <0.0001 \| A-B \| \| Veh vs. Ibr-1 \| -104.9 \| \| -181.7 to -28.05 \| \| Yes \| ** \| 0.0031 \| A-C \| \| Veh vs. Ibr-10 \| -119.1 \| \| -195.9 to -42.31 \| \| Yes \| *** \| 0.0006 \| A-D \| \| Veh vs. Ibr-1 \| 134.3 \| \| 57.53 to 211.1 \| \| Yes \| **** \| <0.0001 \| B-C \| \| Veh vs. Ibr-10 \| 120.1 \| \| 43.27 to 196.9 \| \| Yes \| *** \| 0.0005 \| B-D \| \| Ibr-1 vs. Ibr-10 \| -14.26 \| \| -91.07 to 62.55 \| \| No \| ns \| 0.9621 \| C-D \| |
| **Supplementary Figure 3A. Number of Iba-1 positive cell in the hippocampal CA1** |
| \| Table Analyzed \| \| Iba-1 cell number \| \| \| --- \| --- \| --- \| --- \| \| Data sets analyzed \| \| F-I \| \| \|  \| \|  \| \| \| ANOVA summary \| \|  \| \| \| F \| \| 15.52 \| \| \| P value \| \| <0.0001 \| \| \| P value summary \| \| **** \| \| \| Significant diff. among means (P < 0.05)? \| \| Yes \| \| \| R square \| \| 0.3385 \| \| \| Number of families \| 1 \| \|  \| \|  \|  \|  \|  \| \| Number of comparisons per family \| 6 \| \|  \| \|  \|  \|  \|  \| \| Alpha \| 0.05 \| \|  \| \|  \|  \|  \|  \| \|  \|  \| \|  \| \|  \|  \|  \|  \| \| Tukey's multiple comparisons test \| Mean Diff. \| \| 95.00% CI of diff. \| \| Significant? \| Summary \| Adjusted P Value \|  \| \| Veh vs. Veh \| -314.3 \| \| -439.3 to -189.3 \| \| Yes \| **** \| <0.0001 \| F-G \| \| Veh vs. Ibr-1 \| -156.4 \| \| -281.4 to -31.35 \| \| Yes \| ** \| 0.0080 \| F-H \| \| Veh vs. Ibr-10 \| -85.19 \| \| -211.6 to 41.17 \| \| No \| ns \| 0.2971 \| F-I \| \| Veh vs. Ibr-1 \| 157.9 \| \| 32.94 to 283.0 \| \| Yes \| ** \| 0.0073 \| G-H \| \| Veh vs. Ibr-10 \| 229.1 \| \| 102.8 to 355.5 \| \| Yes \| **** \| <0.0001 \| G-I \| \| Ibr-1 vs. Ibr-10 \| 71.17 \| \| -55.19 to 197.5 \| \| No \| ns \| 0.4573 \| H-I \| |
| **Supplementary Figure 3A. Number of Iba-1 positive cell in the hippocampal DG** |
| \| Table Analyzed \| \| Iba-1 cell number \| \| \| --- \| --- \| --- \| --- \| \| Data sets analyzed \| \| K-N \| \| \|  \| \|  \| \| \| ANOVA summary \| \|  \| \| \| F \| \| 15.41 \| \| \| P value \| \| <0.0001 \| \| \| P value summary \| \| **** \| \| \| Significant diff. among means (P < 0.05)? \| \| Yes \| \| \| R square \| \| 0.3369 \| \| \| Number of families \| 1 \| \|  \| \|  \|  \|  \|  \| \| Number of comparisons per family \| 6 \| \|  \| \|  \|  \|  \|  \| \| Alpha \| 0.05 \| \|  \| \|  \|  \|  \|  \| \|  \|  \| \|  \| \|  \|  \|  \|  \| \| Tukey's multiple comparisons test \| Mean Diff. \| \| 95.00% CI of diff. \| \| Significant? \| Summary \| Adjusted P Value \|  \| \| Veh vs. Veh \| -335.6 \| \| -481.9 to -189.2 \| \| Yes \| **** \| <0.0001 \| K-L \| \| Veh vs. Ibr-1 \| -216.0 \| \| -362.3 to -69.66 \| \| Yes \| ** \| 0.0012 \| K-M \| \| Veh vs. Ibr-10 \| -42.84 \| \| -190.8 to 105.1 \| \| No \| ns \| 0.8730 \| K-N \| \| Veh vs. Ibr-1 \| 119.6 \| \| -26.78 to 265.9 \| \| No \| ns \| 0.1489 \| L-M \| \| Veh vs. Ibr-10 \| 292.7 \| \| 144.8 to 440.6 \| \| Yes \| **** \| <0.0001 \| L-N \| \| Ibr-1 vs. Ibr-10 \| 173.2 \| \| 25.24 to 321.1 \| \| Yes \| * \| 0.0150 \| M-N \| |
| **Supplementary Figure 3B. Number of GFAP positive cell in the Cortex** |
| \| Table Analyzed \| GFAP cell number \| \| \| --- \| --- \| --- \| \| Data sets analyzed \| A-D \| \| \|  \|  \| \| \| ANOVA summary \|  \| \| \| F \| 188.5 \| \| \| P value \| <0.0001 \| \| \| P value summary \| **** \| \| \| Significant diff. among means (P < 0.05)? \| Yes \| \| \| R square \| 0.8601 \| \| \| Number of families \| 1 \|  \| \|  \|  \|  \|  \| \| Number of comparisons per family \| 6 \|  \| \|  \|  \|  \|  \| \| Alpha \| 0.05 \|  \| \|  \|  \|  \|  \| \|  \|  \|  \| \|  \|  \|  \|  \| \| Tukey's multiple comparisons test \| Mean Diff. \| 95.00% CI of diff. \| \| Significant? \| Summary \| Adjusted P Value \|  \| \| Veh vs. Veh \| -440.2 \| -494.7 to -385.8 \| \| Yes \| **** \| <0.0001 \| A-B \| \| Veh vs. Ibr-1 \| -374.4 \| -428.8 to -319.9 \| \| Yes \| **** \| <0.0001 \| A-C \| \| Veh vs. Ibr-10 \| -385.0 \| -439.5 to -330.6 \| \| Yes \| **** \| <0.0001 \| A-D \| \| Veh vs. Ibr-1 \| 65.82 \| 11.37 to 120.3 \| \| Yes \| * \| 0.0112 \| B-C \| \| Veh vs. Ibr-10 \| 55.19 \| 0.7458 to 109.6 \| \| Yes \| * \| 0.0457 \| B-D \| \| Ibr-1 vs. Ibr-10 \| -10.63 \| -65.08 to 43.82 \| \| No \| ns \| 0.9563 \| C-D \| |
| **Supplementary Figure 3B. Number of GFAP positive cell in the hippocampal CA1** |
| \| Table Analyzed \| \|  \| GFAP cell number \| \| \| --- \| --- \| --- \| --- \| --- \| \| Data sets analyzed \| \|  \| F-I \| \| \|  \| \|  \|  \| \| \| ANOVA summary \| \|  \|  \| \| \| F \| \|  \| 25.97 \| \| \| P value \| \|  \| <0.0001 \| \| \| P value summary \| \|  \| **** \| \| \| Significant diff. among means (P < 0.05)? \| \|  \| Yes \| \| \| R squared \| \|  \| 0.4585 \| \| \| Number of families \|  \| 1 \| \|  \| \|  \|  \|  \|  \| \| Number of comparisons per family \|  \| 6 \| \|  \| \|  \|  \|  \|  \| \| Alpha \|  \| 0.05 \| \|  \| \|  \|  \|  \|  \| \|  \|  \|  \| \|  \| \|  \|  \|  \|  \| \| Tukey's multiple comparisons test \|  \| Mean Diff. \| \| 95.00% CI of diff. \| \| Below threshold? \| Summary \| Adjusted P Value \|  \| \| - vs. - \|  \| -360.9 \| \| -468.4 to -253.4 \| \| Yes \| **** \| <0.0001 \| F-G \| \| - vs. 1 \|  \| -182.1 \| \| -289.6 to -74.57 \| \| Yes \| *** \| 0.0001 \| F-H \| \| - vs. 10 \|  \| -210.2 \| \| -317.7 to -102.7 \| \| Yes \| **** \| <0.0001 \| F-I \| \| - vs. 1 \|  \| 178.8 \| \| 71.29 to 286.3 \| \| Yes \| *** \| 0.0002 \| G-H \| \| - vs. 10 \|  \| 150.7 \| \| 43.16 to 258.2 \| \| Yes \| ** \| 0.0023 \| G-I \| \| 1 vs. 10 \|  \| -28.12 \| \| -135.6 to 79.38 \| \| No \| ns \| 0.9028 \| H-I \| |
| **Supplementary Figure 1B. Number of GFAP positive cell in the hippocampal DG** |
| \| Table Analyzed \| \| GFAP cell number \| \| \| --- \| --- \| --- \| --- \| \| Data sets analyzed \| \| K-N \| \| \|  \| \|  \| \| \| ANOVA summary \| \|  \| \| \| F \| \| 52.79 \| \| \| P value \| \| <0.0001 \| \| \| P value summary \| \| **** \| \| \| Significant diff. among means (P < 0.05)? \| \| Yes \| \| \| R squared \| \| 0.6325 \| \| \| Number of families \| 1 \| \|  \| \|  \|  \|  \|  \| \| Number of comparisons per family \| 6 \| \|  \| \|  \|  \|  \|  \| \| Alpha \| 0.05 \| \|  \| \|  \|  \|  \|  \| \|  \|  \| \|  \| \|  \|  \|  \|  \| \| Tukey's multiple comparisons test \| Mean Diff. \| \| 95.00% CI of diff. \| \| Below threshold? \| Summary \| Adjusted P Value \|  \| \| - vs. - \| -400.7 \| \| -486.6 to -314.8 \| \| Yes \| **** \| <0.0001 \| K-L \| \| - vs. 1 \| -286.4 \| \| -372.3 to -200.5 \| \| Yes \| **** \| <0.0001 \| K-M \| \| - vs. 10 \| -215.4 \| \| -301.3 to -129.5 \| \| Yes \| **** \| <0.0001 \| K-N \| \| - vs. 1 \| 114.3 \| \| 28.39 to 200.2 \| \| Yes \| ** \| 0.0042 \| L-M \| \| - vs. 10 \| 185.3 \| \| 99.40 to 271.2 \| \| Yes \| **** \| <0.0001 \| L-N \| \| 1 vs. 10 \| 71.01 \| \| -14.90 to 156.9 \| \| No \| ns \| 0.1415 \| M-N \| |

**References**

[1] H.J. Lee, and H.S. Hoe, Inhibition of CDK4/6 regulates AD pathology, neuroinflammation and cognitive function through DYRK1A/STAT3 signaling. Pharmacol Res 190 (2023) 106725.

[2] H.J. Lee, J.H. Park, and H.S. Hoe, Idebenone Regulates Abeta and LPS-Induced Neurogliosis and Cognitive Function Through Inhibition of NLRP3 Inflammasome/IL-1beta Axis Activation. Front Immunol 13 (2022) 749336.
